# Room-temperature skyrmion lattice in a layered magnet (Fe_0.5_Co_0.5_)_5_GeTe_2_

**DOI:** 10.1126/sciadv.abm7103

**Published:** 2022-03-23

**Authors:** Hongrui Zhang, David Raftrey, Ying-Ting Chan, Yu-Tsun Shao, Rui Chen, Xiang Chen, Xiaoxi Huang, Jonathan T. Reichanadter, Kaichen Dong, Sandhya Susarla, Lucas Caretta, Zhen Chen, Jie Yao, Peter Fischer, Jeffrey B. Neaton, Weida Wu, David A. Muller, Robert J. Birgeneau, Ramamoorthy Ramesh

**Affiliations:** 1 Department of Materials Science and Engineering, University of California, Berkeley, Berkeley, CA, USA.; 2 Materials Sciences Division, Lawrence Berkeley National Laboratory, Berkeley, CA, USA.; 3 Physics Department, University of California, Santa Cruz, Santa Cruz, CA, USA.; 4 Department of Physics and Astronomy, Rutgers University, Piscataway, NJ, USA.; 5 School of Applied and Engineering Physics, Cornell University, Ithaca, NY, USA.; 6 Department of Physics, University of California, Berkeley, Berkeley, CA, USA.; 7 Department of Electrical Engineering, University of California, Berkeley, Berkeley, CA, USA.; 8 Department of Chemistry, University of California, Berkeley, Berkeley, CA, USA.; 9 Kavli Energy Nanosciences Institute at Berkeley, Berkeley, CA, USA.; 10 Kavli Institute at Cornell for Nanoscale Science, Cornell University, Ithaca, NY, USA.

## Abstract

Novel magnetic ground states have been stabilized in two-dimensional (2D) magnets such as skyrmions, with the potential next-generation information technology. Here, we report the experimental observation of a Néel-type skyrmion lattice at room temperature in a single-phase, layered 2D magnet, specifically a 50% Co–doped Fe_5_GeTe_2_ (FCGT) system. The thickness-dependent magnetic domain size follows Kittel’s law. The static spin textures and spin dynamics in FCGT nanoflakes were studied by Lorentz electron microscopy, variable-temperature magnetic force microscopy, micromagnetic simulations, and magnetotransport measurements. Current-induced skyrmion lattice motion was observed at room temperature, with a threshold current density, *j*_th_ = 1 × 10^6^ A/cm^2^. This discovery of a skyrmion lattice at room temperature in a noncentrosymmetric material opens the way for layered device applications and provides an ideal platform for studies of topological and quantum effects in 2D.

## INTRODUCTION

Two-dimensional van der Waals (2D vdW) magnets offer an excellent platform for exploring fascinating magnetic and quantum topological phases, owing to their unique layered structure and crystal symmetries. (*1*–*5*). Magnetic skyrmions, which are topologically protected spin textures, are often observed in chiral (*6*–*8*) and polar (*9*–*14*) magnets and are usually stabilized by an antisymmetric exchange interaction, the Dzyaloshinskii-Moriya interaction (DMI). Recent investigations of 2D vdW magnets have led to the discovery and manipulation of skyrmions, a promising new frontier for future spintronic devices (*15*–*19*). Néel-type skyrmions supported by interfacial DMI energy were found in the WTe_2_/Fe_3_GeTe_2_ (*17*) and O-Fe_3_GeTe_2_/Fe_3_GeTe_2_ (*18*) heterostructures. In addition, zero-field Néel-type skyrmions were stabilized by interlayer exchange coupling between Fe_3_GeTe_2_ and Co/Pd multilayers (*19*). However, these previously reported vdW skyrmions were all achieved below room temperature, limiting the further investigation of their formation mechanism and subsequent potential developments toward room-temperature applications. The discovery of an intrinsic magnetic ground state, especially a topological skyrmion state, at room temperature in layered materials provides an ideal platform to study the interfacial coupling between spin and other degrees of freedom, which may give rise to novel quantum behaviors at room temperature. Here, we report the realization of a room-temperature Néel-type skyrmion lattice in 50% Co–doped Fe_5_GeTe_2_ (FCGT), which is a layered polar ferromagnetic metal. Its static properties and dynamic behavior of ~85- to 2100-nm-thick nanoflakes were characterized using Lorentz (scanning) transmission electron microscopy [L(S)TEM], variable-temperature magnetic force microscopy (MFM) for domain imaging supported by micromagnetic simulations. Magnetotransport measurements were performed to understand the spin dynamics of the skyrmion lattice. Our studies demonstrate the dependence of skyrmion size and stability of the skyrmion lattice as a function of sample thickness, applied magnetic field, and temperature, as well as the current-induced skyrmion formation and motion at room temperature.

## RESULTS AND DISCUSSION

### Structure and room-temperature skyrmions of the FCGT system

**Fig. 1.**
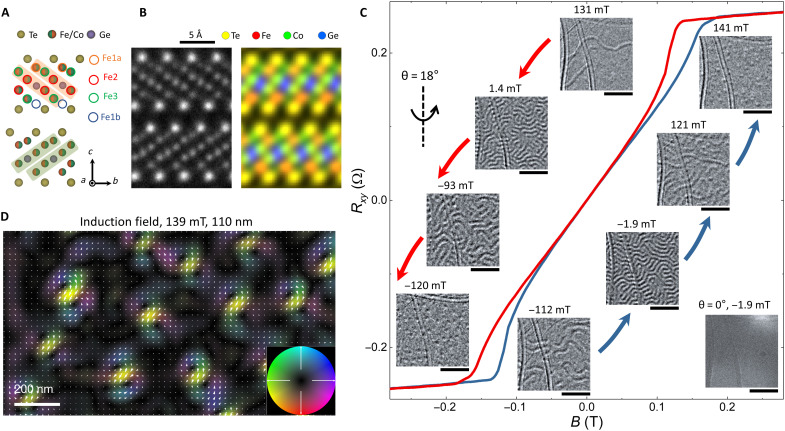
Skyrmion creation as a function of the out-of-plane magnetic field at room temperature. (**A**) Structural model of FCGT viewed along the *a* axis. Fe1a site, Fe2 site, Fe3 site, and Fe1b site are marked by orange, red, green, and blue circles, respectively. (**B**) Experimental atomic resolution HAADF-STEM image (left) and the corresponding STEM–energy-dispersive x-ray spectroscopy (EDS) map (right). The STEM-EDS map is shown as a composite overlay of Fe, Co, Ge, and Te with red, green, blue, and yellow color maps, respectively. (**C**) Hysteresis curve of a 110-nm-thick FCGT flake and the corresponding Lorentz TEM images at various applied fields. The red (blue) curve indicates the direction for decreasing (increasing) magnetic fields. The contrast change between 0° and 18° tilt reveals the Néel character. Scale bars, 500 nm. (**D**) Magnetic induction field map of isolated skyrmions in a 110-nm-thick flakes obtained using 4D-LSTEM along with an EMPAD, with applied fields of 139 mT. The color and arrows indicate induction field components perpendicular to the beam propagation direction of Neél skyrmions at an 18° tilt.

The AA′-stacked FCGT is a polar ferromagnetic metal that belongs to the *P*6_3_*mc* space group and *C*_6*v*_ point group. Figure 1A shows a schematic of FCGT with the wurtzite structure. High-angle annular dark-field–based STEM (HAADF-STEM) images (Fig. 1B and fig. S1) show the atomic-scale structure of FCGT and the high quality of our single-crystalline FCGT nanoflakes. Similar to Fe_5_GeTe_2_, the FGCT unit cell nominally features three unique Fe Wyckoff positions: Fe1, Fe2, and Fe3; Fe1 is further split by fractional occupation between the Fe1a and Fe1b sites (Fig. 1, A and B). Unlike the undoped Fe_5_GeTe_2_ system where the partially occupied Fe1 positions are disordered (*20*–*23*), Fe1 atoms in the AA′-stacked FCGT system are ordered, and six layers [Fe/Co (Fe1a site), Fe/Co (Fe3 site), Fe/Co (Fe2 site), Ge, Fe/Co (Fe2), and Fe/Co (Fe3)] are separated by two adjacent Te layers. The crystal structure sublayers exhibit an ordered zigzag arrangement along the *c* axis (and hence the notation of AA′) as viewed from the [100] projection. In addition, Co (Fe) atoms prefer to occupy the Fe2 (Fe3) site in the AA-stacked FCGT (45%) (*22*); however, most of the Co (Fe) atoms still occupy the Fe2 (Fe3) site, but a fraction of Co (Fe) atoms occupy the Fe3 (Fe2) site in the AA′-stacked FCGT (see Fig. 1B and fig. S2). Thus, the AA′-stacked FCGT system is a unique noncentrosymmetric structure in the Fe*_N_*GeTe_2_ (*N* = 3, 4, and 5) system (*22*, *24*, *25*). A notable feature arising as a result of the pronounced order of the two Fe1 sites in the AA′ phase is the absence of an inversion center located at the Ge site that is observed in the bulk AA-stacked Fe_5_GeTe_2_. While the inversion point disallows any cumulative DM vector beyond the unit cell, thus restricting any long-range chiral spin texture in Fe_3_GeTe_2_ (*26*), the DM interactions in the AA′ FCGT are far less constrained and become sensitive to the extent of the ordering of two Fe1 site occupancies. Application of Moriya’s symmetry rules (*27*) to the nearest-neighbor spins in the AA′ FCGT system restricts all antisymmetric interactions to be oriented in plane and transverse to the pair axis, yet in contrast to the AA phase, the AA′ phase with ordered Fe1 site occupation allows for an uncompensated DMI vector to persist beyond the atomic scale. Given the broken inversion symmetry and the expected in-plane DMI of the AA′ phase, one expects to observe Néel-type skyrmions in this system, in contrast to most bulk DMI systems (such as the B20 compounds), which exhibit only Bloch-type skyrmions (*28*, *29*).

The temperature dependence of magnetization and magnetotransport (see figs. S3 and S4) reveals an unexpectedly high Curie temperature for this FCGT system, up to ~350 K. The sheared out-of-plane anomalous Hall transport curves at room temperature indicate a multidomain state at remanence (*13*), which hints that a skyrmion might be stabilized under a suitable magnetic field in this polar magnet. L(S)TEM measurements were carried out at room temperature to resolve the nanoscale spin texture of FCGT. Magnetic field–dependent L(S)TEM images are shown in Fig. 1C, superimposed on the anomalous Hall resistance plot from a sample of similar thickness. At *B* = −1.9 mT, no contrast in the L(S)TEM image is observed for the 110-nm-thick FCGT nanoflake when the sample is not tilted; that is, the electron beam direction is collinear with the normal to the sample’s surface. Once the sample is slightly tilted (for example, by 18°) along the direction labeled in Fig. 1C, the labyrinthine domain is observed, which suggests a Néel-type character of the domain wall (*30*–*32*). The absence of contrast in the L(S)TEM image for the nontitled sample is due to the symmetric distribution of magnetization in the Néel-type domain wall, leading to an electron deflection that cancels out. At field magnitudes beyond 200 mT, no contrast can be observed, which is indicative of a uniform (single domain) magnetized state. As the magnetic field is progressively decreased toward zero field (red curve), stripe domains start to appear (131 mT) and progressively aggregate to form worm-like features (1.4 mT), but skyrmions are hardly observed under this field. However, with a further decrease of the magnetic field and the subsequent change of the field direction, the stripe domains turn into a mixture of short stripe domains and bubbles (−93 mT) and then evolve into bubbles (−120 mT). The bubbles show a dark/bright contrast along the direction perpendicular to the tilted axis, which further confirms the Néel-type spin texture in this system. For field magnitudes beyond ~−200 mT, the image contrast is uniform, indicating a uniform magnetic state. Recovering a skyrmionic bubble state requires that the field be progressively increased in the positive direction to about 130 to 140 mT (see also movie S1). Figure 1D shows a magnetic induction map obtained using 4D Lorentz STEM (4D-LSTEM) in conjunction with an electron microscope pixel array detector (EMPAD) (*33*). The theoretical simulations and experimental results demonstrate that the Néel-type skyrmion can be described by a magnetic induction composed of clockwise and counterclockwise spin curls in the induction field map (*31*, *34*), which also agrees well with our experimental observation in Fig. 1D and the corresponding simulation in fig. S5.

### Thickness controlled skyrmion size and ordering

**Fig. 2.**
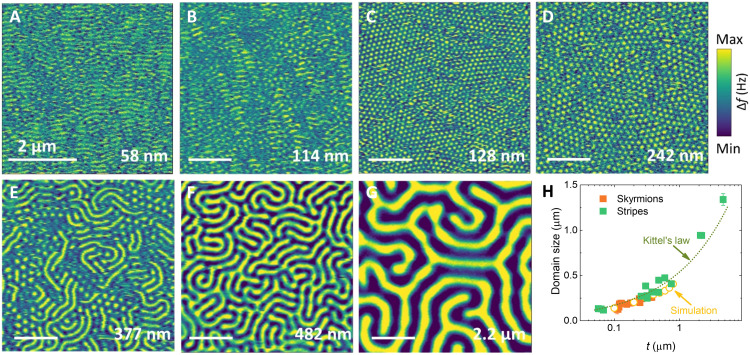
MFM measurements at room temperature. (**A** to **G**) MFM images of an FCGT nanoflake on a SiO_2_/Si substrate with different thicknesses (scale bar, 2 μm). The thickness was measured by atomic force microscopy. (**H**) Domain size as a function of the FCGT thickness. The skyrmion size strongly depends on flake thickness. The dotted line is a fit to Kittel’s law. The micromagnetic simulation results also follow Kittel’s law (yellow circles).

For Bloch-type skyrmions, the lateral size is independent of the sample thickness, as exemplified in the B20 compound (*35*, *36*). By contrast, Neél-type skyrmion sizes can be extensively manipulated by the thickness of the sample owing to magnetostatic interactions (*37*). FCGT, as a layered material, can be systematically exfoliated into nanoflakes with varying thickness, starting from few unit cells. To study the evolution of the skyrmions as a function of sample thickness, MFM measurements were performed at room temperature and zero magnetic field. The nanoflakes were prepared by mechanical exfoliation from bulk crystals and were capped with a 3-nm-thin Au layer to prevent degradation. Figure 2 (A to G) depicts MFM images of the FCGT nanoflakes with various thicknesses, where yellow (blue) contrast corresponds to spin up (down) regions. Stripe domains were observed in a 58-nm-thick nanoflake (Fig. 2A). As the thickness of the FCGT nanoflake increases to ~114 nm (Fig. 2B), isolated patches of bubbles (skyrmions) emerge in the stripe domains. As the thickness is increased to ~128 nm, an ordered hexagonal skyrmion lattice emerges (Fig. 2C). The skyrmion lattice is stable over a broad thickness range until the nanoflake is thicker than ~300 nm. For thicknesses greater than ~300 nm, a mixture of isolated skyrmion and labyrinthine domains was observed (Fig. 2E), and a further thickness increase gradually stabilizes the labyrinthine domain state, as exemplified in the 482-nm-thick (Fig. 2F) and 2.2-μm-thick (Fig. 2G) nanoflakes. No magnetic contrast was observed in a nanoflake of thickness below ~40 nm; however, it is likely that the magnetic signal falls below the MFM sensitivity because of the reduced stray field in thin nanoflakes (*38*). Figure 2H shows the thickness-dependent domain width and skyrmion size, which are determined from the line profile of the stripes and bubbles. The domain size diminishes with smaller thickness, following Kittel’s law (*d* ∝ *t*^1/2^, where *d* is the domain size and *t* is the sample thickness). This is also verified by micromagnetic simulations illustrated in Fig. 2H. From these measurements and simulations, we estimated a DMI energy of *D* = 0.76 ± 0.02 mJ/m^2^ (see section S1).

### Metastable zero-field skyrmions

**Fig. 3.**
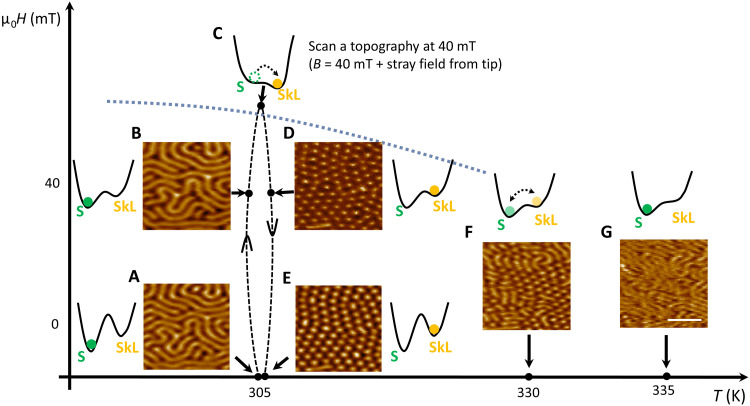
Metastable zero-field skyrmions. MFM images in the 261-nm FCGT nanoflake and the corresponding schematic *H*-*T* energy landscape diagram of the stripe domain state (S) and skyrmion lattice state (SkL). (**A** and **B**) Stripe domain state at 0 and 40 mT, at 305 K. (**C**) The skyrmion lattice phase was induced by the combination of the external field (40 mT) and the stray field from the MFM tip. (**D** and **E**) Skyrmion lattice state persists as a metastable state at 40 and 0 mT. (**F**) Coexistence of stripes and skyrmions at 330 K. (**G**) Fully recovered stripe domain state at 335 K. No magnetic contrast was observed above 340 K. The dotted line is the phase boundary between stripe domain and skyrmion lattice. Scale bar, 1 μm.

We now discuss the possible mechanisms that lead to the observation of a zero-field skyrmion lattice in the FCGT nanoflakes. In previous works, zero-field Néel-type skyrmions have been stabilized by an exchange bias field (*39*), stray fields from MFM tips (*40*, *41*), and Joule heating (*42*–*44*) in multilayer ferromagnetic metal films. The exchange bias scenario can be eliminated because anomalous Hall measurements on zero-field skyrmion samples exhibit no measurable shift after either positive or negative field cooling (see fig. S4). A metastable skyrmion lattice can be stabilized at low temperature by quenching from a thermally equilibrated skyrmion lattice phase near the transition temperature (*T*_c_) (*42*, *43*). It is worth noting that the *T*_c_ of bulk FCGT is just above room temperature, and thus, it is ideal to stabilize skyrmions at room temperature. The reduced *M*_s_ near *T*_c_ favors the formation of skyrmion due to the reduced energy barriers from stray fields in nanoflakes (*43*). Last, local fields from the scanning MFM tip can also be the driving force to create the nominally zero-field skyrmion lattice (*45*). To investigate the origin of the creation of a zero-field skyrmion lattice in FCGT, a series of MFM measurements under different conditions were carried out. Figure 3 demonstrates that the metastable skyrmion lattice phase can be stabilized in zero field through field training. At zero field, the stripe domain phase (which is the ground state) is observed after zero field cooling through *T*_c_ (Fig. 3A). So, the free energy of the skyrmion lattice phase is higher, likely because of the positive energy cost of domain walls (see the ‘Micromagnetic simulations’ section). The Zeeman energy gain of the skyrmion lattice phase due to a finite, out-of-plane magnetic field lowers its free energy, resulting in a transition from stripe domains to a skyrmion lattice. At room temperature, the transition field to induce skyrmion lattice is >100 mT (see Lorentz TEM results in Fig. 1C). For the same reason, the stray field from the MFM tip (several tens of milliteslas as shown in fig. S6) by itself is not sufficient to induce a skyrmion lattice phase at room temperature (see fig. S7). At 305 K (slightly above room temperature), the stripe phase is stable in a 40-mT external field (Fig. 3B). This, in combination with the several tens of milliteslas field from the MFM tip, can induce a skyrmion lattice (Fig. 3D), further validating that the combined field is above the transition field (Fig. 3C). The induced skyrmion lattice is stable in a 40-mT field at 305 K as shown in Fig. 3D, suggesting a substantial energy barrier between the two phases. Furthermore, the skyrmion lattice phase persists as a metastable state even after removing the external field (Fig. 3E), corroborating the substantial energy barrier. The skyrmion lattice phase is robust against a mild temperature increase (up to 312 K). A mild thermal annealing even improves the correlation length of the metastable skyrmion lattice (see fig. S8). However, further increases in temperature destabilize the skyrmion lattice phase, which gradually returns to the stripe domains through a mixed state (Fig. 3F), indicating the gradual decrease of the energy barrier due to the reduction of *M*_s_ and magnetic anisotropy with *T*_c_. The stripe phase completely recovers at ~335 K in zero field (Fig. 3G).

### Micromagnetic simulations

**Fig. 4.**
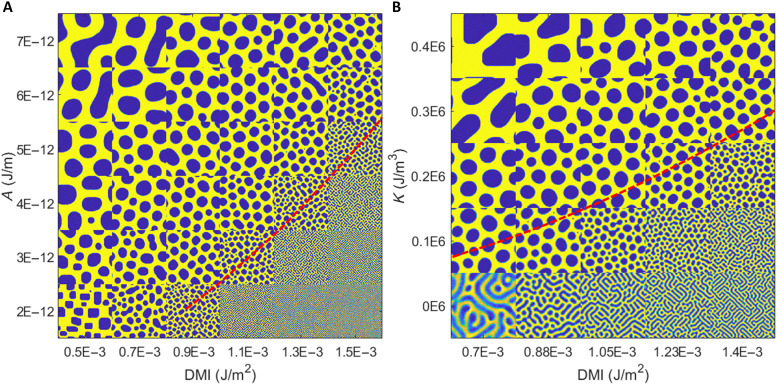
Micromagnetic simulations. (**A**) Skyrmion phase diagram as a function of DMI constant *D* and exchange stiffness constant *A* in 200-nm FCGT. (**B**) Skyrmion phase diagram as a function of DMI constant *D* and effective PMA energy *K*. We have used a 4-nm maximum distance between unit cells, which is lower than the value of the exchange length $l_{\text{ex}}=\sqrt{\frac{2A}{u_{o}M_{s}^{2}}\sim }$ 9.8 nm. The image size is 1 μm by 1 μm. The red dashed line follows the solution of σ*_w_* = 0.

To better understand the stabilization of the zero-field skyrmion lattice and its evolution as a function of key magnetic parameters [DMI constant *D*, effective perpendicular magnetic anisotropy (PMA) energy *K*, and exchange stiffness constant *A*] in the FCGT system, micromagnetic simulations were performed as a function of thickness at room temperature (see Methods). During the simulation process, a temporary 100-mT out-of-plane magnetic field was applied to nucleate the skyrmion lattice at room temperature. The skyrmion lattice remains even after removing the field, which is consistent with the MFM experimental results. Figure 4 summarizes the micromagnetic simulations that demonstrate a metastable skyrmion lattice in the 200-nm FCGT nanoflake for certain combinations of *A*, *K*, and *D*. These simulations reveal that the skyrmionic spin texture results from the competition between the magnetic dipolar energy and domain wall energy. For a fixed thickness of the nanoflake and temperature, the domain pattern tends to be progressively dependent on the domain wall energy (*46*) ($\sigma_{w}=4\sqrt{\mathrm{AK}}-\pi |D|$). In the high-*D* and low-*A* (*K*) regime, i.e., σ*_w_* < 0, a labyrinthine domain pattern with a small domain width is observed at zero field. An external magnetic field is needed to stabilize the skyrmion lattice. In contrast, large bubbles and stripe domains were obtained in the high-*A* (*K*) and low-*D* regime (high σ*_w_*). For σ*_w_* in the range of ~0 to 1.62 mJ/m^2^, a skyrmion lattice can be stabilized in a 200-nm-thick nanoflake, as confirmed by experimental data (see section S1). Similar to the results with 200-nm-thick nanoflakes, the domain pattern in 50- and 100-nm-thick nanoflakes also transforms from a labyrinthine domain pattern to a skyrmion lattice and returns to a labyrinthine domain with a large width, and lastly to a single domain with increasing σ*_w_*. The skyrmion lattice phase was observed only in a very narrow range of σ*_w_* for the relative thin nanoflake, which is attributed to a larger dipolar energy (see fig. S11). Even under a magnetic field, the skyrmions are more difficult to be stabilized in the thinner nanoflake as evidenced by the L(S)TEM measurement. With increasing field, the labyrinthine domain at remanence in a 60-nm-thick nanoflake reverses via rapid domain wall motion through the entire sample (see fig. S12). On the other hand, when the nanoflakes are thick, the skyrmion lattice can be stabilized under a magnetic field (see fig. S13A). However, breaking up of the larger-width labyrinthine domain into bubbles requires overcoming a large energy barrier. Therefore, a metastable skyrmion lattice can be readily stabilized at zero magnetic field only for nanoflakes with thicknesses between 120 and 300 nm.

### Current-driven spin dynamics of skyrmions

**Fig. 5.**
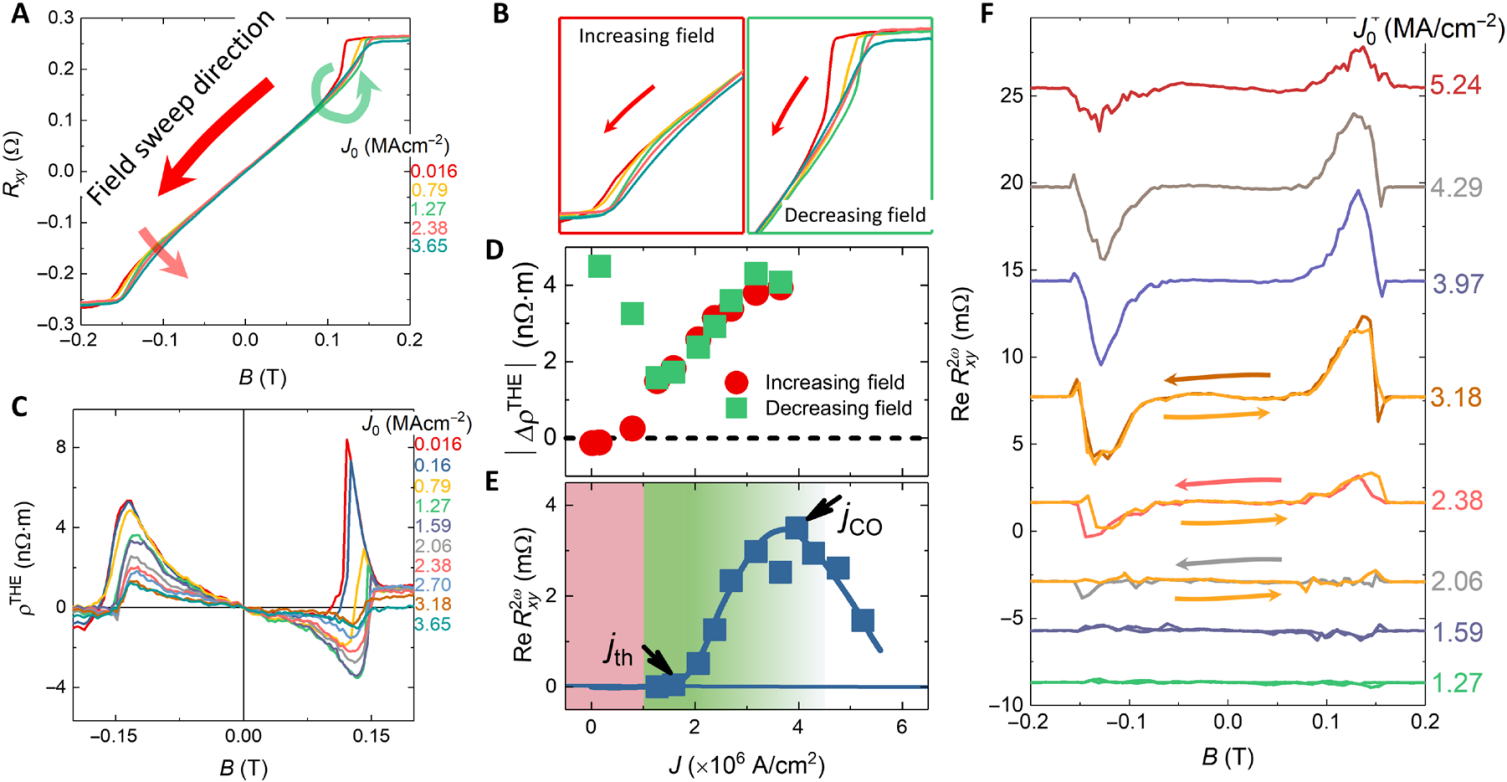
Room-temperature current-driven spin dynamics of skyrmions of FCGT nanoflake. (**A**) Hall measurements at various current densities. (**B**) Zoom-in of the Hall curve near saturated field. (**C**) Magnetic field dependence of topological Hall resistivity at various current densities. Current density dependence of ∣∆ρ^THE^∣ (**D**) and Re $R_{\mathrm{xy}}^{2\omega }$ (**E**). (**F**) Magnetic field–dependent Re $R_{\mathrm{xy}}^{2\omega }$ at various current densities.

Now that the stabilization of a skyrmion lattice at room temperature is verified, to further demonstrate the potential of the FCGT system for skyrmionic devices, we performed the magnetotransport measurements to study the current-driven motion of skyrmions. Figure 5A shows Hall measurements at various current densities in a 136-nm-thick nanoflake at room temperature. The sample temperature was monitored by the longitudinal resistance of the sample itself to eliminate the heating effect (see fig. S14). The Hall resistance consists of the normal, anomalous, and topological Hall resistances. The topological Hall effect has been associated with a noncollinear spin texture that comprises the skyrmions (*47*). At low current density, the Hall effect curves show a sheared out-of-plane hysteresis loop with two distinct slopes, i.e., sharp (decreasing field) and slanted (increasing field) slopes (labeled by green and red arrows). From the L(S)TEM results (Fig. 1 and movie S1), the skyrmions are stabilized only when the field magnitude is increased from ~−120 to ~−140 mT. As the current density is increased, a suppression in the Hall effect curve (marked by a red arrow in Fig. 5A and a red box in Fig. 5B) is observed when increasing the field. In prior work, such a current-dependent suppression of the Hall resistance has been explained by the progressive decrease of the topological Hall resistance. The current-induced skyrmion motion is expected to cause an “emergent” electric field perpendicular to the direction of motion, which opposes the topological Hall field, leading to a reduction of the measured topological Hall resistance (*48*–*50*).

The topological Hall curve (see Methods) was calculated as shown in Fig. 5C. Using the peak value of the ρ^THE^ in Fig. 5C as a representative measure of the topological Hall component, its dependence on the current density, i.e., the differential topological Hall resistivity ∣Δρ^THE^∣, was studied. This current density dependence of the ∣Δρ^THE^∣ is shown in Fig. 5D. Focusing on the data plotted in red circles, one can see that below the threshold current density, *j*_th_, of ~1×10^6^ A/cm^2^ for skyrmion motion, the stationary skyrmions are pinned by defects, and therefore, the topological Hall resistivity remains constant. Above *j*_th_, the skyrmion starts to move, and the topological Hall resistivity decreases. Further increases in the current density do not significantly change Δρ^THE^. From this, the skyrmion drift velocity (*v*) can be estimated to be ~35.4 m/s at *j* ~ 4.76 × 10^6^ A/cm^2^ (*49*) (see fig. S15 and section S2 for the details of this calculation).

When decreasing the field from saturation (labeled by a green arrow in Fig. 5A and a green box in Fig. 5B), there is no skyrmion phase from the L(S)TEM measurements. The sharp peak exhibits a positive value, similar to that in the increasing field regime. The sharp kink is not induced by a topological Hall effect associated with the skyrmions but by a different nucleation process (see Fig. 1C). As the current density increases, the sign of the resistivity in the peak position reverses and the absolute value is close to that when the field increases starting from zero (red circles) (Fig. 5D). This indicates that the skyrmion lattice can be induced by the applied current and the skyrmion density increases with increasing current density, as expected from ∣ρ^THE^∣ ∝ *n*_sk_ (skyrmion density) (*51*).

To further probe the skyrmion dynamics, second-harmonic Hall measurements were performed. In a noncentrosymmetric ferromagnet (*52*, *53*), the electrical resistance generally depends on the current and magnetic field direction (*54*) and thus a nonreciprocal magnetotransport signature is anticipated, originating from an emergent electromagnetic field induced by skyrmion motion (*52*). The spin dynamics of the vortex-like domain can be deduced by the phase diagram as a function of the second-harmonic resistance and the current density, where three distinct regimes were identified (*52*): (i) *j* < *j*_th_ (threshold current density), the real part of the second-harmonic resistance (Re $R_{\mathrm{xy}}^{2\omega }$) is essentially zero, which indicates that the skyrmion lattice is stationary; (ii) *j*_th_ < *j* < *j*_CO_ (a crossover current density), the Re $R_{\mathrm{xy}}^{2\omega }$ monotonically increases because of the disordered motion of the skyrmion lattice; and (iii) *j*_0_ > *j*_CO_, the Re $R_{\mathrm{xy}}^{2\omega }$ decreases, the lattice state turns into a dynamical reordering due to the relative reduction of the pinning force at large current densities. Figure 5F shows the typical profiles of the second-harmonic Hall effect measured at various current densities. The magnitude of Re $R_{\mathrm{xy}}^{2\omega }$ measured with *j*_0_ = 1.27 × 10^6^ A/cm^2^ is small and below the noise floor of the measurements. As the current density reaches 2.06 × 10^6^ A/cm^2^, a peak occurs when increasing the field from zero, while Re $R_{\mathrm{xy}}^{2\omega }$ is almost zero when decreasing the field from a saturated state. This indicates that the skyrmion motion when increasing the fields from zero exhibits a low *j*_th_. In contrast, the back-and-forth curve overlaps well once the measured current density is raised up to *j* = 2.4 × 10^6^ A/cm^2^, indicating that the skyrmion dynamics are similar for both increasing and decreasing fields. The *j*_th_ is ~2.06 × 10^6^ A/cm^2^, which is also in agreement with the topological Hall effect results. When the current density is higher than *j*_CO_ (3.97 × 10^6^ A/cm^2^), Re $R_{\mathrm{xy}}^{2\omega }$ begins to decrease. Figure 5E shows a room-temperature phase diagram as a function of current density and Re $R_{\mathrm{xy}}^{2\omega }$ at peak position. The threshold current density for skyrmion lattice motion at room temperature in the FCGT system is smaller than for a traditional ferromagnetic metal (*13*, *55*–*58*).

### Phase diagram

**Fig. 6.**
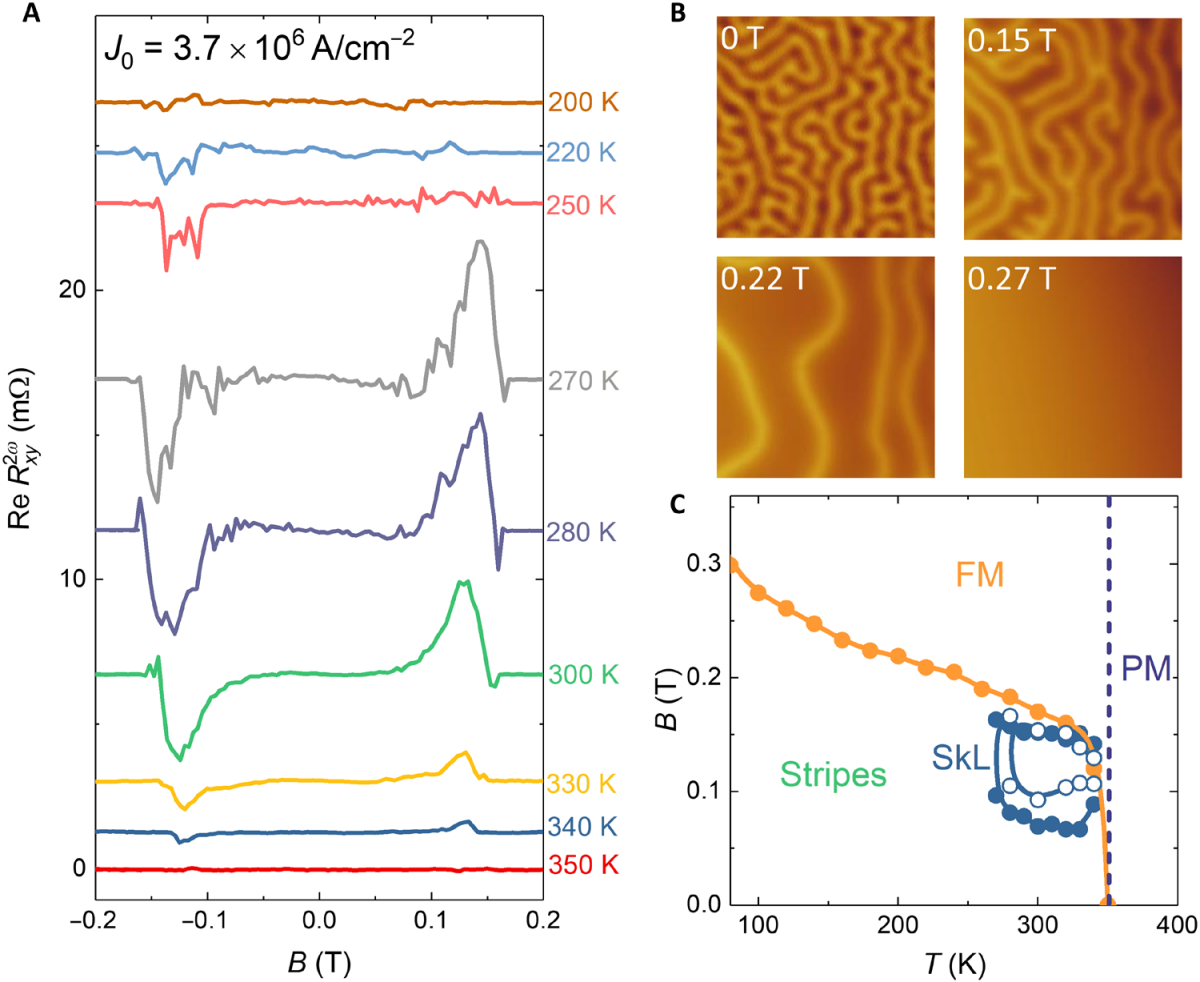
Phase diagram. (**A**) Magnetic field dependence of the real part of second-harmonic Hall resistance at various temperatures. (**B**) MFM images on a 261-nm nanoflake were taken in 0, 0.15, 0.22, and 0.27 T at 200 K, respectively. The color scales of MFM images are 1.6, 5.5, 6, and 0.2 Hz, respectively. The size of the images is 5 μm by 5 μm. The lift height is 150 nm. (**C**) Phase diagram of FCGT as a function of temperature and magnetic field obtained in 136-nm-thick nanoflakes. The blue, green, yellow, and purple areas represent skyrmion lattice (SkL), stripe, ferromagnetic (FM), and paramagnetic phases (PM), respectively. The solid and empty circles correspond to the results of the second-harmonic Hall resistance measured by 3.7 × 10^6^ and 2.7 × 10^6^ A/cm^−2^, respectively.

Figure 6A exhibits magnetic field–dependent Re $R_{\mathrm{xy}}^{2\omega }$ in the FCGT nanoflake with current densities *j*_0_ = 3.7 × 10^6^ A/cm^2^ at various temperatures. Above 340 K, no measurable signal was observed, which is consistent with the temperature-dependent MFM measurements. A strong peak occurs at the temperature range of 270 to 340 K, which indicates that the skyrmion lattice can be stabilized in this temperature regime. As the temperature further decreases, the peak intensity abruptly decreases and hints at the existence of a few isolated skyrmions. This result is further confirmed by the field dependence of MFM measurements at 200 K (see Fig. 6B and fig. S16). At zero field, the MFM image shows a labyrinthine stripe domain structure with equal populations of domains with opposite magnetization; these stripes also show some small branches. With increasing magnetic field, the antiparallel stripe domains reverse one by one rather than by breaking into bubbles. The PMA in the FCGT system rapidly increases as the temperature is decreased and plays a more dominant role compared to the DMI constant and *A* (*59*). A strong PMA helps in decreasing the skyrmion density (*60*), and thus, no skyrmion lattice phase was observed at 200 K under any magnetic field. Last, Fig. 6C compiles the phase stability diagram for the FCGT system as a function of temperature (*T*) and magnetic field (*B*).

In summary, we have demonstrated that the AA′-stacked FCGT is a new class of room-temperature Néel-type skyrmion hosting material with *C*_6*v*_ symmetry and that skyrmion lattices can be stabilized over a wide range of thickness, magnetic field, and temperature, specifically at zero field and room temperature. The skyrmion lattices can be easily manipulated by the stray field of an MFM tip and current density. Current-induced skyrmion lattice formation and motion were achieved at room temperature. We expect that by subtle tuning of the magnetic parameters using chemical doping, stacking with spin-orbit coupling material, ion radiation, etc., one can achieve small skyrmions with high mobility under a low current density. Therefore, our system provides a promising platform for 2D skyrmion-based logic and memory devices, notably at room temperature.

## METHODS

### Sample synthesis

Single crystals of (Fe_0.5_Co_0.5_)_5–δ_GeTe_2_ were grown by the chemical vapor transfer method. Iodine (I_2_) were used as the transport agent to assist the growth of single crystals. Elemental Fe powder (99.99%), Co powder (99.99%), small Ge pieces (99.999%, 1 to 3 mm in size), and Te powder (99.999%) were used for the sample synthesis. The starting raw materials were fully mixed together with the nominal molar ratio Fe:Co:Ge:Te = 3:3:1:2 inside the glovebox. About 30 mg of iodine was added to the mixture. The starting mixture was then vacuumed, back-filled with one-third volume of argon, and sealed inside the quartz tube with an inner diameter of 8 mm, an outer diameter of 12 mm, and a length of about 150 mm. The sealed quartz tube was placed horizontally inside a muffle furnace during the growth. The reaction temperature was set to 750°C under isothermal conditions for up to 7 or 10 days. Small single crystals were harvested by quenching the furnace at 750°C in air. Excess iodine was removed from the surfaces of the single crystals with ethanol.

### Scanning transmission electron microscopy

Cross-sectional TEM specimens were prepared on the FCGT nanoflakes, using a Thermo Fisher Helios G4 UX focused ion beam with a Ga^+^ ion beam of 30 and 5 keV, and followed by a final milling step of 2 keV to reduce damage. To protect the surface from ion beam damage, we deposited carbon and platinum layers before milling. The thickness of the cross-sectional TEM specimen was determined to be ~10 nm by convergent beam electron diffraction analysis. HAADF-STEM images were acquired by using a Cs-corrected FEI Titan operated at 300 keV, with a beam semiconvergence angle of 21.4 mrad and a beam current of 10 pA.

The atomic-resolution energy-dispersive x-ray spectroscopy (EDS) mapping was acquired with a Thermo Fisher Scientific “Kraken” Spectra 300 operated at 120 kV equipped with Dual-X EDS detectors with a collection solid angle of 1.76 sr. The STEM-EDS maps were acquired with a probe current of 120 pA and a semiconvergence angle of 30 mrad, continuously raster-scanned with drift correction with a dwell time of 2 μs and over a total acquisition time of ~4 hours. The specimen thickness was ~10 nm estimated by convergent beam electron diffraction analysis. The elemental maps were first obtained by fitting the peak intensities above the background with the Cliff-Lorimer method. Nonlocal principal components analysis was used to reduce the Poisson noise in the obtained EDS elemental maps, which further revealed the low-frequency details of atomic features (*61*).

### Micromagnetic simulation

Micromagnetic simulations were performed using the graphical processing unit–accelerated finite mesh package MuMax3 (*62*). To reproduce the experimentally observed spin textures, simulations were run over a phase space of material parameters close to the experimentally measured values. The parameter scans are presented in Fig. 4 as mosaics where each tile is a 1 μm by 1 μm rendering of the *z* component of magnetization from a single simulation.

Simulations incorporated the dipole-dipole interaction and finite temperature. Periodic boundary conditions were used because the simulated area 1 μm by 1 μm is smaller than the actual sample. The parameters in the Hamiltonian are exchange constant *A*, first-order uniaxial anisotropy *K*, saturation magnetization *M*_s_, and interfacial DMI constant *D*. The cell size of 3.9 nm by 3.9 nm by 3.1 nm is below the exchange length $l_{\text{ex}}=\sqrt{\frac{2A}{u_{o}M_{s}^{2}}\sim }$ 9.8 nm.

Figure 2H explores the Kittel effect in terms of skyrmion diameter dependence on sample thickness at zero field. The simulation area is 1 μm by 1 μm by (100 to 900) nm. A cell size of 3.9 nm by 3.9 nm by 3.1 nm is used. In these simulations *A* = 4 × 10^−12^ J/m, *K* = 0.24 × 10^6^ J/m^3^, *D* = 0.9 mJ/m^2^, *M*_s_ = 301 kA/m, and *T* = 293 K. The *M*_s_ and *K*_u_ are estimated by the isothermal magnetization curves of the bulk FCGT at room temperature. The simulation is initialized in a skyrmion lattice state with a skyrmion diameter of 60 nm. The initial state is relaxed using the MuMax3 standard relax function. The simulation is then advanced using a time evolver for 10 ns to incorporate thermal effects.

Figure 4 explores the phase space of spin textures over varying material parameters. In Fig. 4, the simulation area is 1 μm by 1 μm by 200 nm. A cell size of 3.9 nm by 3.9 nm by 3.1 nm is used. In these simulations, *A* = (2 × 10^−12^ to 7 × 10^−12^) J/m, *K* = (0.0 × 10^6^ to 0.4 × 10^6^) J/m^3^, *D* = (0.5 to 1.5) mJ/m^2^, *M*_s_ = 301 kA/m, and *T* = 293 K. When held constant, *A* = 4 × 10^−12^ J/m and *K* = 0.24 × 10^6^ J/m^3^. The simulation is initialized in a random state with a 100-mT magnetic field oriented along the normal axis. The initial state is relaxed using the MuMax3 standard relax function. The field is then removed, and the simulation is advanced using a time evolver for 10 ns to reach the zero-field state and incorporate thermal effects.

Figure S11 explores the phase space of spin textures over varying material parameters and sample thicknesses. In fig. S11, the simulation area is 1 μm by 1 μm by (50 to 200) nm. A cell size of 3.9 by nm 3.9 by nm 3.1 nm is used. In these simulations, *A* = (2 × 10^−12^ to 7 × 10^−12^) J/m, *K* = (0.0 × 10^6^ to 0.4 × 10^6^) J/m^3^, *D* = (0.7 to 1.4) mJ/m^2^, *M*_s_ = 301 kA/m, and *T* = 293 K. When held constant, *A* = 4 × 10^−12^ J/m and *K* = 0.24 × 10^6^ J/m^3^. The simulation is initialized in a random state with a 100-mT magnetic field oriented along the normal axis. The initial state is relaxed in the applied field using the MuMax3 standard relax function. The field is then removed, and the simulation is advanced using a time evolver for 10 ns to reach the zero-field state and incorporate thermal effects.

### MFM measurement

The thickness-dependent room-temperature MFM measurements were performed using an Asylum Research MFP-3D system. The MFM images (color bar, yellow and blue) were measured by typical tapping/lift mode. The lift height is fixed at 100 nm for scanning nanoflakes of thickness above 100 nm to reduce the influence of stray fields created by the MFM tips.

The temperature-dependent MFM measurements (Figs. 3 and 6B and fig. S16) (color bar, brown and white) were performed in a homebuilt variable-temperature MFM (Rutgers University) using commercial piezoresistive cantilevers with a spring constant *k* ~ 3 N/m and a resonant frequency *f*_0_ ~ 43 kHz. The tips were coated with nominally 100-nm Co by using magnetron sputtering. The MFM signal (the shift of resonant frequency) is proportional to the out-of-plane stray field gradient, which was extracted by a phase-locked loop (SPECS). MFM images were taken with constant-height noncontact mode. The magnetic field was applied by clamping two permanent magnets on the side of MFM probe. The north poles of two magnets face each other to generate an out-of-plane field (max, ~0.04 T) with a tiny in-plane field component (<0.003 T) due to misalignment, calibrated by a gauss meter. The cryogenic MFM measurements were performed inside a superconducting magnet.

### Transport measurement

FCGT nanoflakes were exfoliated onto SiO_2_/Si substrates using a scotch tape. Polymethyl methacrylate (PMMA) is spin-coated on the substrate, and then e-beam lithography is used to pattern the samples. After development in a commercial solution (KAYAKU MIBK/IPA 1:3), we deposit 3-nm Cr/100-nm Au to form electrodes by means of e-beam evaporation. Acetone eventually removes the PMMA resist and the residual Cr/Au. Following the abovementioned process, high-quality Hall bar devices were fabricated on the basis of FCGT nanoflakes.

Magnetoresistance and Hall resistance were performed in a cryogen-free measurement system from Cryogenic Ltd. with the applied DC currents of 100 μA. The skyrmion dynamic measurements were obtained using the standard lock-in technique (SR-830, Stanford Research Systems) with AC modulated at 23 Hz. Given the increase in sample temperature owing to Joule heating, we did not use the temperature controller from the transport measurement system to monitor the temperature of the samples. We derived the temperature from the longitudinal resistance of the nanoflake sample itself and adjusted the environment temperature of the sample so that the sample temperature remained at room temperature. The topological Hall resistivity can be derived from the Hall resistance and isothermal magnetization curve; however, the magnetization of nanoflake is too small to measure via bulk magnetic characterization. Therefore, to obtain a rough estimate of the topological Hall resistance, we assume that the topological Hall resistivity is negligible under a large current density (~4 ×10^6^ A/cm^2^). Under this assumption, the topological Hall resistivity can be estimated as the difference between the Hall resistivity under the measured current density and saturated current density.

## Supplementary Materials

### This PDF file includes:

Sections S1 and S2 Figs. S1 to S16 References
