# Supplementary material for: Room-temperature skyrmion lattice in a layered magnet (Fe0.5Co0.5)5GeTe2
Source: Sci Adv. 2022 Mar 23;8(12):eabm7103. doi: 10.1126/sciadv.abm7103 (PMC8942374; doi:10.1126/sciadv.abm7103)
Supplement: Supplementary file 1 — Sections S1 and S2 Figs. S1 to S16 References [file sciadv.abm7103_sm.pdf]

Science Advances
AAAS

Supplementary Materials for

Room-temperature skyrmion lattice in a layered magnet $(Fe_{0.5}Co_{0.5})_5GeTe_2$

Hongrui Zhang*, David Raftrey, Ying-Ting Chan, Yu-Tsun Shao, Rui Chen, Xiang Chen, Xiaoxi Huang, Jonathan T. Reichanadter, Kaichen Dong, Sandhya Susarla, Lucas Caretta, Zhen Chen, Jie Yao, Peter Fischer, Jeffrey B. Neaton, Weida Wu, David A. Muller, Robert J. Birgeneau, Ramamoorthy Ramesh*

*Corresponding author. Email: hongruizhang@berkeley.edu (H.Z.); rramesh@berkeley.edu (R.R.)

Published 23 March 2022, *Sci. Adv.* **8**, eabm7103 (2022)
DOI: 10.1126/sciadv.abm7103

The PDF file includes:

Sections S1 and S2
Figs. S1 to S16
Legend for movie S1
References

Other Supplementary Material for this manuscript includes the following:

Movie S1

Section S1. Estimation of DMI constant, D

The relationship between domain size and domain wall energy can be written as(*63*):

$$\frac{\sigma_w}{\mu_0 M_s^2 t} = \frac{d^2}{t^2} \sum_{odd\ n=1}^{\infty} \frac{1}{(\pi n)^3} \left[1 - \left(1 - \frac{2\pi n t}{d}\right) exp\left(\frac{-2\pi n t}{d}\right)\right]$$

Thus, we find that the domain wall energy σ_w is essentially constant for a thickness much larger than the dipolar length (l_w~13.4 nm, $l_w = \frac{\sigma_w}{\mu_0 M_s^2}$, where σ_w is domain wall energy, M_s =301.6 kA/m is saturated magnetization; the average value of σ_w is ~1.5 mJ/m^2. σ_w is described by(*46*) : $\sigma_w = 4\sqrt{AK} - \pi|D|$,where A is the exchange stiffness constant (estimated value of $A = 4\times10^{-12}$ J/m at room temperature from the micromagnetic simulation), K is the effective perpendicular anisotropy energy ($K = 2.4\times10^5$ J/m^3), and D is the DMI constant. From these measurements, we calculated the DMI constant $D = 0.76 \pm 0.02$ mJ/m^2, the value of which is also relatively unchanged with thickness.

Section S2. Estimation of skyrmion drift velocity

The skyrmion drift velocity (v) was calculated by the equation(*49*):

$$v = jR_0\left(1 - \rho_{xy}^{THE}(j)/\rho_{xy}^{THE}(j \leq j_c)\right)$$

where j is current density, R_0 is the normal Hall coefficient. Fig. S15 shows the velocity of skyrmion as a function of the current density. At j ~ 4.76 MA/cm^2, v is ~ 35.4 m/s which is larger than that of O-Fe_3GeTe_2/Fe_3GeTe_2 bilayer vdW materials at 100 K, ~ 1 m/s (18) and smaller than that of multilayer ferro/ferrimagnetic thin films at room temperature,~ 50 m/s(*64*).

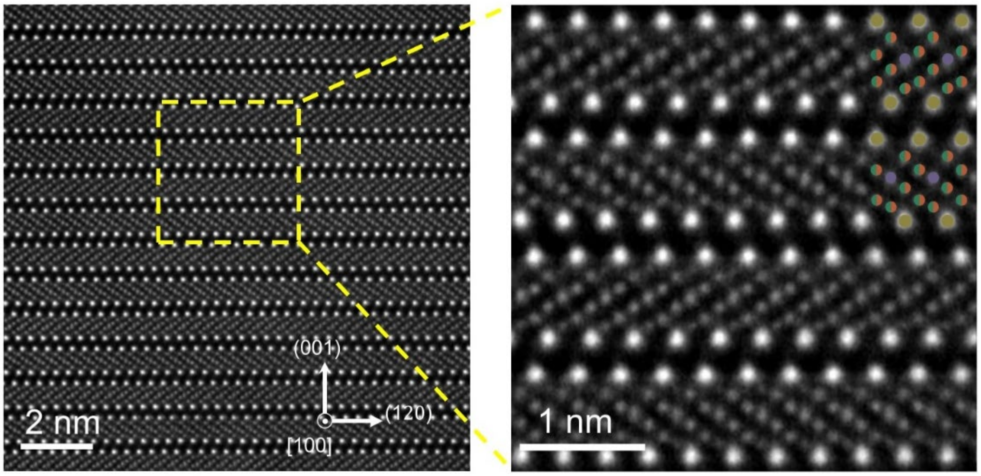

Fig. S1 Atomic structure of the FCGT system. Corresponding HAADF-STEM cross-sectional images of FCGT along the [100] direction.

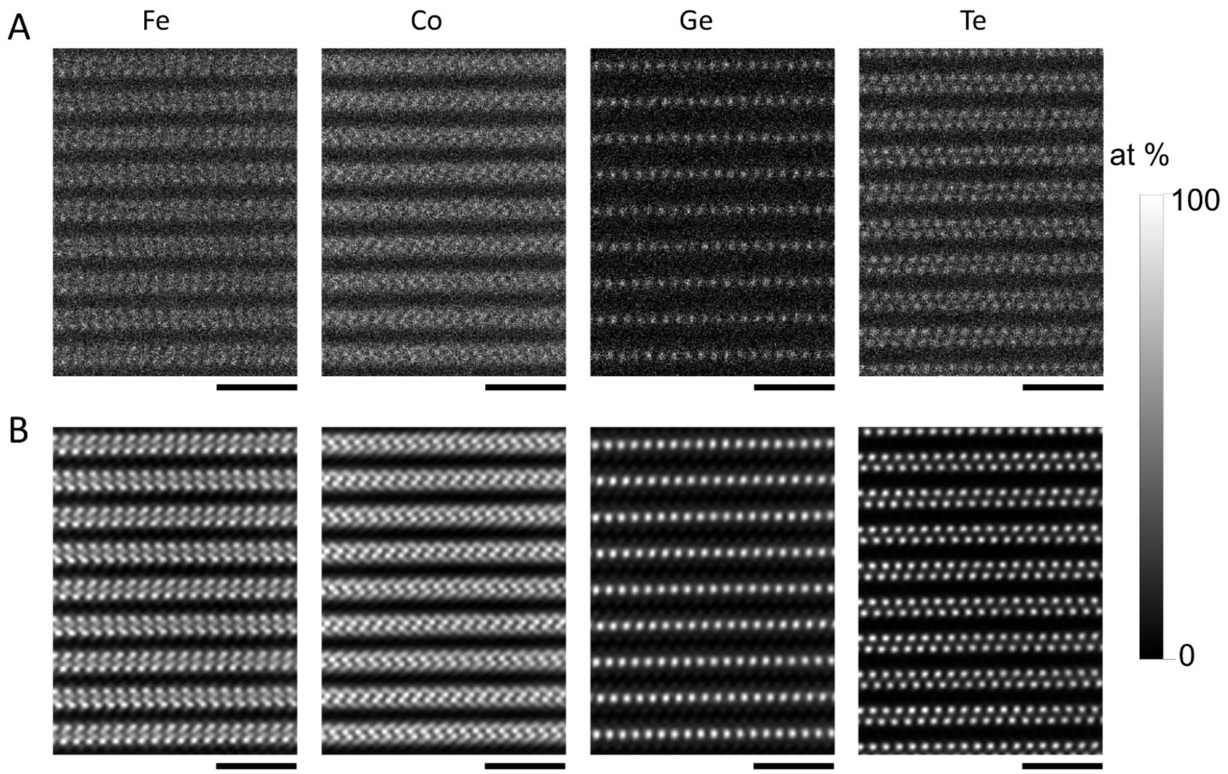

Fig. S2 STEM-EDS map of the FCGT sample. (**A**) Left to right: raw STEM-EDS maps for Fe, Co, Ge, Te were shown in atomic percent, respectively. The spectra were fitted with Gaussian peaks above the background, followed by analysis using the Cliff-Lorimer method. (**B**) EDS maps with non-local PCA denoising. Scale bars, 2 nm.

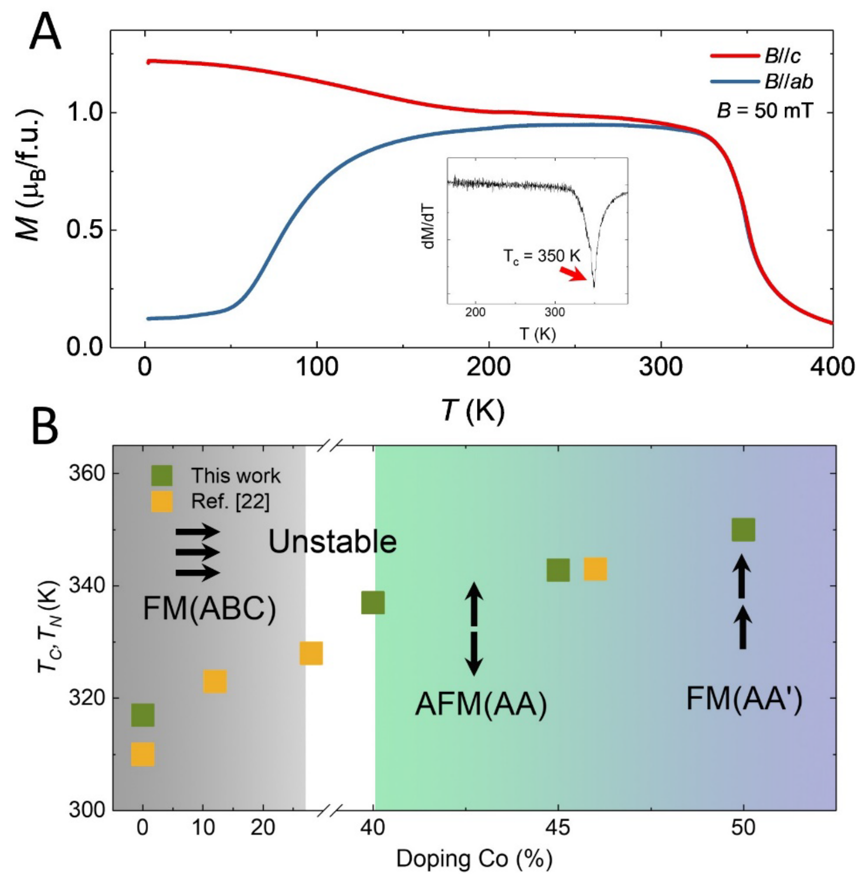

Fig. S3 Magnetic property of FCGT system. (**A**) Temperature-dependent magnetization of FCGT (50%) bulk at a magnetic field of 50 mT along the *ab* plane and *c* axis. The inset shows the first derivative of the magnetization as a function of temperature. The red arrow exhibits the ferromagnetic-paramagnetic transition indicates the Curie temperature is about 350 K. (**B**) Magnetic phase diagram as a function of Co doping level.

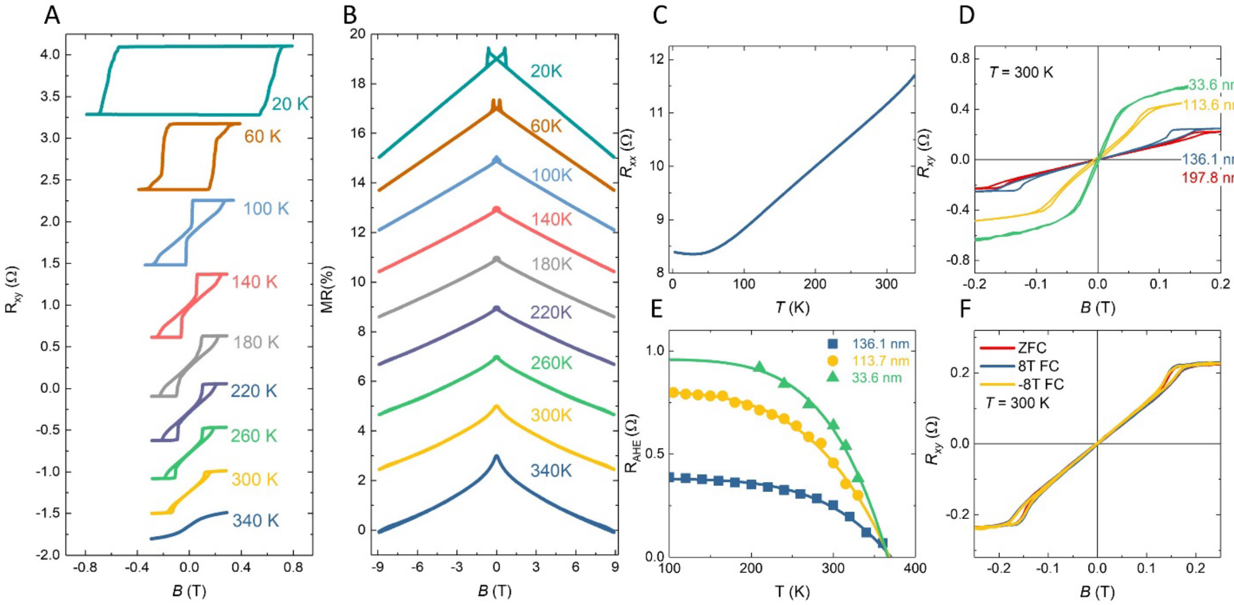

Fig. S4 Transport measurements of the FCGT nanoflake. Anomalous Hall effect (**A**) and magnetoresistance (**B**) curves of a 136.1-nm-thick nanoflake at various temperatures suggest that the FCGT system is in a ferromagnetic ground state. In the 260~340 K range, the anomalous Hall effect curves show a sheared out-of-plane hysteresis loop which indicates a multidomain state at remanence. The stripe domain can easily break into the skyrmion bubble, then gradually shrink with the field increasing, which results in the slow increase of anomalous Hall resistance. In the 100~220K range, the stripe domain reverses via rapid domain wall motion through the entire sample (see Fig. S16), which brings about a relatively sharp loop. When the temperature is below 60 K, rectangular-shaped loops are observed, indicating a single domain state at remanence. The resistance-temperature curve, which shows a metallic behavior, is shown in Fig. S4**C**. Figure S4**D** and **E** show the magnetic field-dependent anomalous Hall resistance at room temperature and temperature dependence of the saturated anomalous Hall resistance for various thicknesses. The AA phase FCGT is an antiferromagnetic ground state. The Néel temperature is above room temperature. Thus, an exchange bias should be observed in the AA' phase FCGT with the AA phase disorder. Anomalous Hall effect curves of a 197.8-nm-thick nanoflake after 0, +8 T, and -8 T field cooling from 400 K show no exchange bias in this system. (Fig. S4**F**) This result indicates that the nanoflake is a pure AA' phase FCGT without the AA phase disorder.

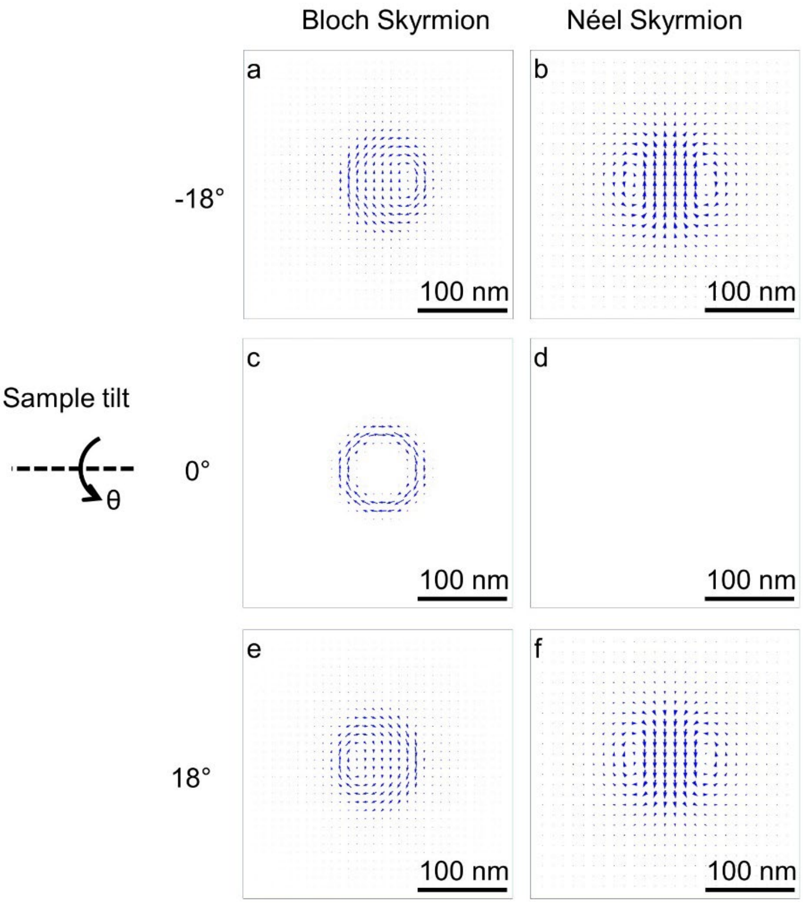

Fig. S5 Simulated magnetic induction field from Bloch-type and Néel-type Skyrmions at different sample tilt angles. Magnetic induction field distribution for a (**A,C,E**) Bloch skyrmion and a (**B,D,F**) Néel skyrmion at tilt angles of -18°, 0°, and -18° were calculated from magnetization distribution of an isolated skyrmion generated using the 360° domain wall model(*65-67*). Since the phase gradient obtained from L(S)TEM is proportional to the induction field, the "biskyrmion" shape is a signature of a Néel-type skyrmion observed at a tilted angle. The simulation was carried out using the skyrmion diameter of 90 nm, domain wall width of 4.2 nm, saturated magnetization M_S of 301.6 kA/m, and sample thickness of 110 nm. The slight shape difference between simulations and experiments can be explained by the distorted skyrmion shape (elongation) resulting from the experimentally applied external magnetic field.

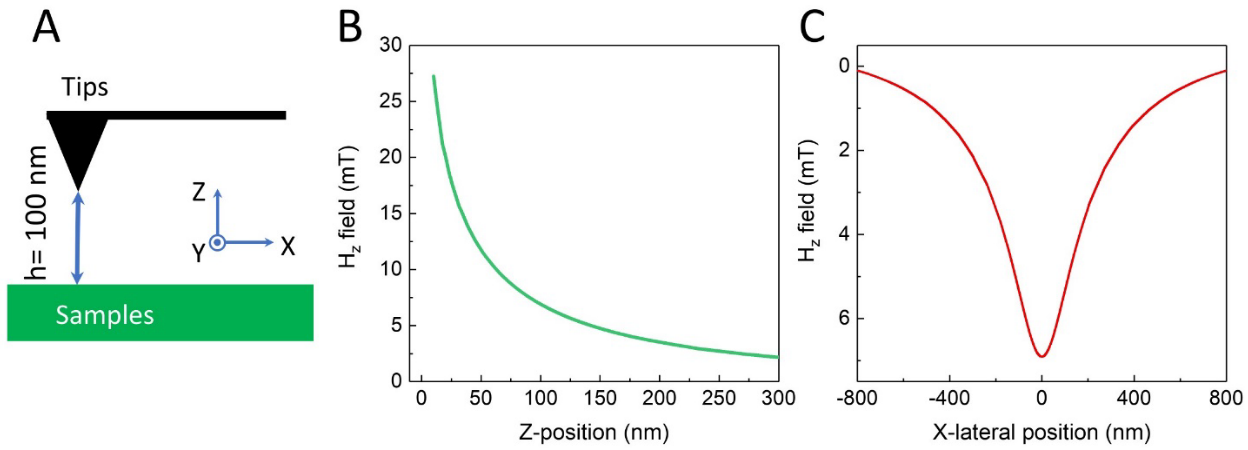

Fig. S6 Estimation of stray fields of MFM tips. The magnetic field around the AFM tip is estimated using COMSOL, where a finite element model of both the AFM tip and the magnetic field around the tip is established. The input parameters are based on the pyramid-shaped silicon AFM tip with a 100-nm-thick hard magnetic coating. The magnetization of the coating film is set to be 300 kA/m. Figure S6**B** shows the Z-position along the tip dependent on the H_z fields. Figure S6**C** shows the X-lateral position at 100 nm height dependent on the H_z fields. The H_z field at 100 nm below the tip is about 7 mT.

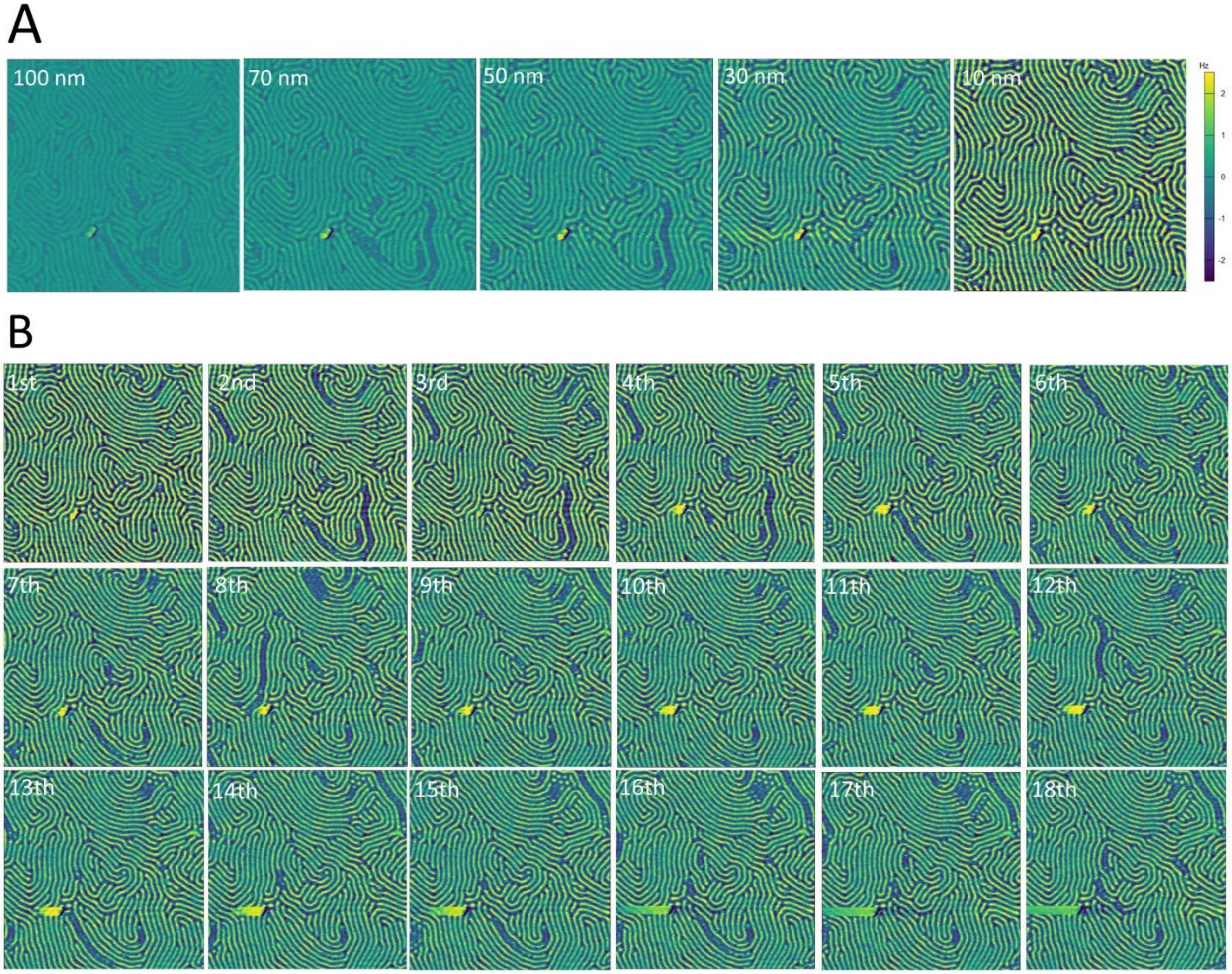

Fig. S7 MFM images at different scanning conditions. (**A**) MFM images measured at different lift heights. (**B**) MFM images were repeated 18 times in the same region. The lift height is fixed at 10 nm. Note that the original state of the sample shows a room temperature zero-field skyrmion. After heating the sample to above T_c, it was cooled down to room temperature. The domain pattern transformed from the skyrmions to the stripe domain. During MFM measurements, the tips will induce a small stray field. In our system, based on anomalous Hall effect results, the skyrmion phase for the 110~2100 nm nanoflake can be stabilized when the magnetic field is between 0.1~0.2 T. Obviously, the stray field is far below this value (27 mT, at 10-nm lift height). The stripe domain almost remains no matter how many scanning times, as shown in Fig. S7**B**.

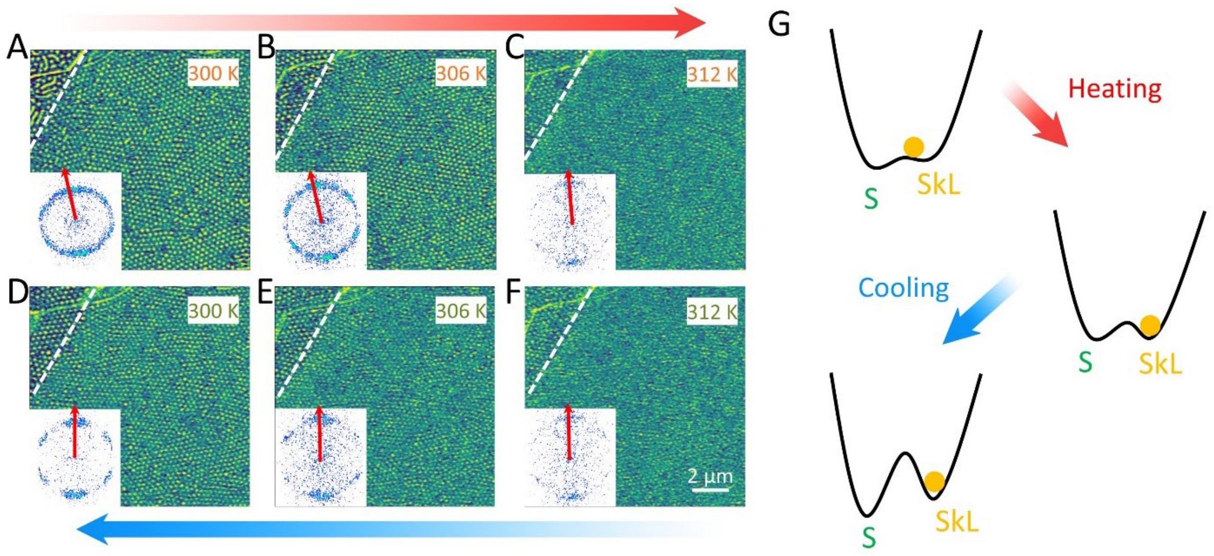

Fig. S8 Spin texture of FCGT nanoflakes at varying temperatures. Temperature-dependent MFM images of 242-nm- (A-F) thick nanoflake. The triangle in the upper left corner in (**A**-**F**) shows the results of a 300-nm thick nanoflake. The insets of the figure show the fast Fourier transform (FFT) of the skyrmion lattices. In the initial state, the skyrmion lattice in the 242-nm-thick nanoflake is stabilized at room temperature and zero field. The FFT (inset of Fig. S8**A**) exhibiting an oval shape indicates the relatively low symmetry of the skyrmion lattice, corresponding to a liquid phase. (*68*) As the temperature increases up to 312 K, the skyrmion size shrinks, and the skyrmion lattice parameter also decreases, indicating that the density of skyrmions is increasing. The long-range order of the skyrmion lattice is enhanced in FFT (Fig. S8**B**) which corresponds to a hexatic phase. (*68*) The hexagonal order increases with temperature (*69*) which may be due to the low nucleation energy for the formation of a skyrmion lattice and decreasing pinning effect at a higher temperature. In addition, the scanning by MFM is beneficial for the formation of an ordered hexagonal lattice. When the samples are heated up to 320 K, the skyrmions are annihilated, and then the samples are then gradually cooled down to room temperature. After the cooling process, the skyrmion sizes and lattice parameters are slightly smaller than in the initial states. However, the hexatic phase is retained. The upper-left corner in Fig. S8 **A**-**F** shows a similar measurement of results for the 300 nm nanoflake. As the temperature increases, the stripe domain is broken into bubbles. After the thermal cycling, an ordered skyrmion lattice was obtained. The stripe contrast in the top right corner (which is an artifact induced by the bump of the surface) doesn't change at all temperatures.

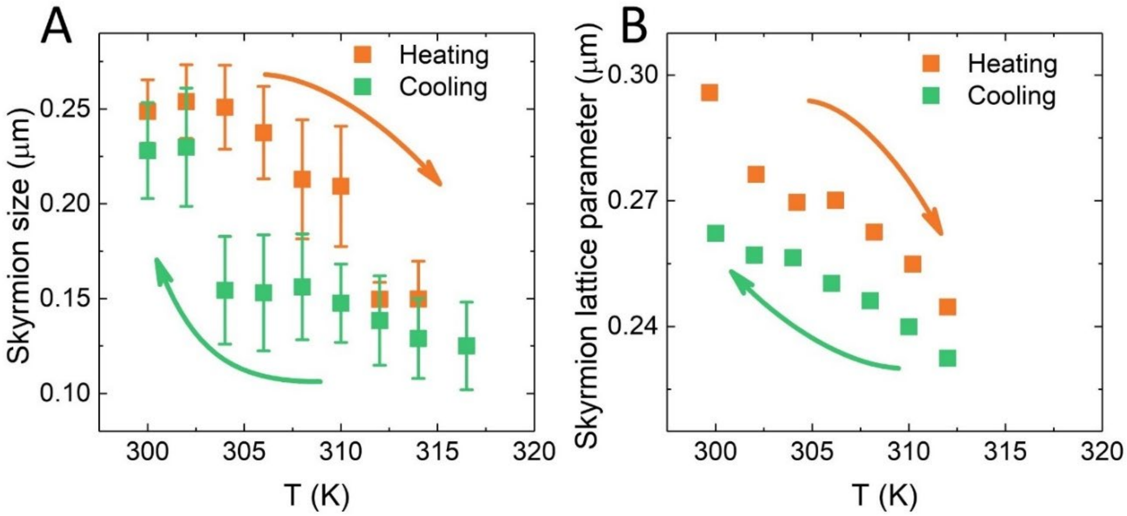

Fig. S9 Temperature-dependent skyrmion size and lattice parameter. As the temperature increases, the skyrmion size (**A**) and lattice parameter (**B**) decrease. Both values are slightly smaller than in the initial state, which may be due to the relaxation of the skyrmion pinned by defects.

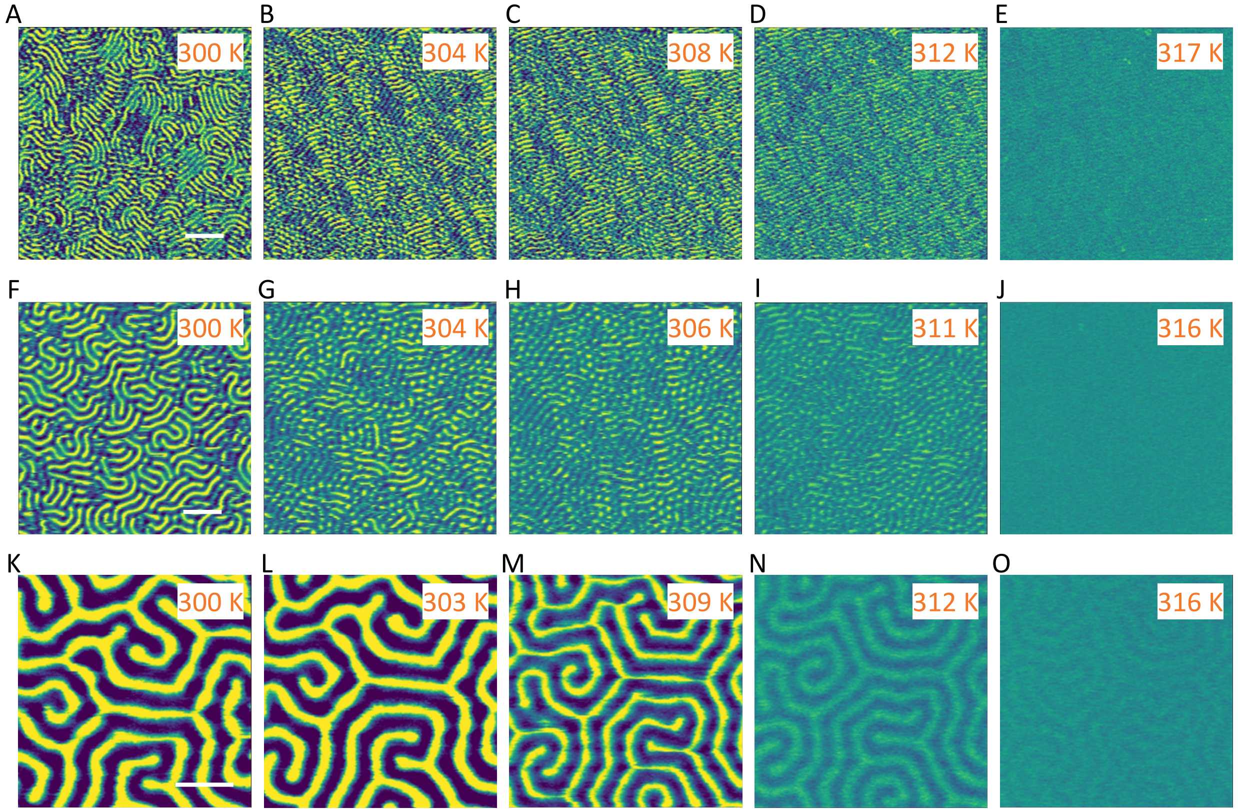

Fig. S10 Temperature-dependent spin texture for different thickness nanoflakes. Temperature-dependent MFM images at zero magnetic field for different thickness nanoflakes, 323 nm (A-E), 471 nm (F-J), and 2.2 μm (K-O). The scale bar is 2 μm.

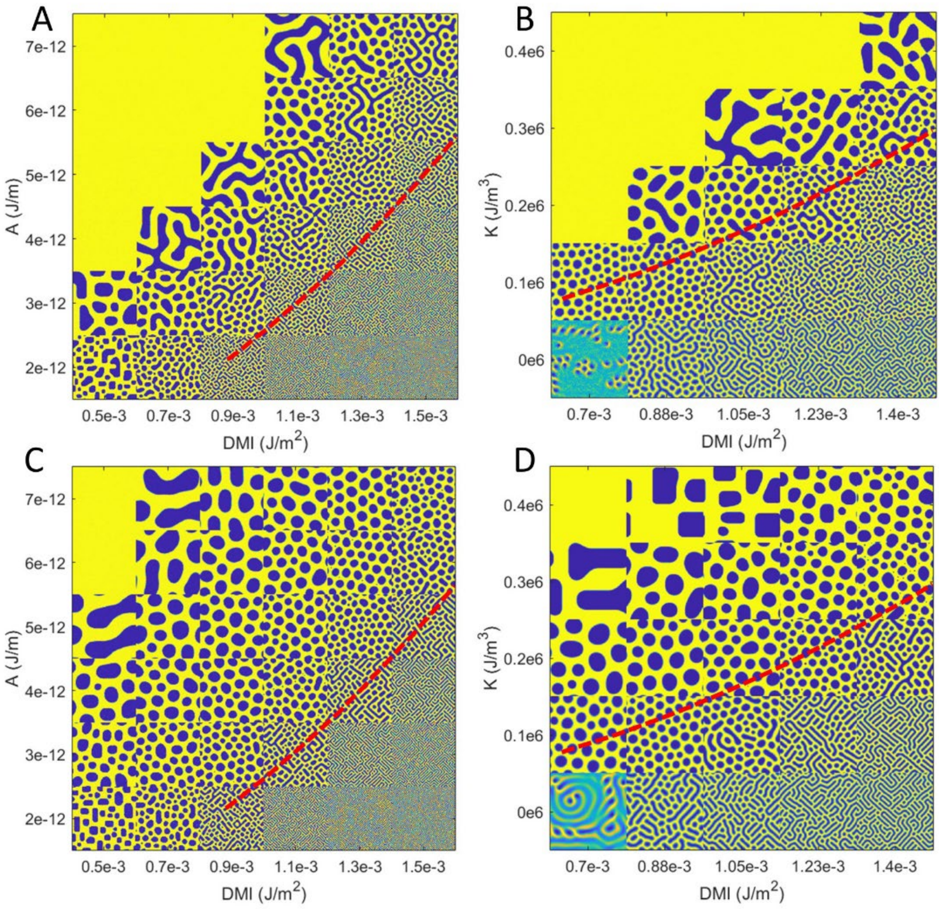

Fig. S11 Micromagnetic simulations. Skyrmion phase diagram of 50-nm-thick (**A**) and 100-nm-thick (**C**) FCGT nanoflake as a function of *D* and *A*. Skyrmion phase diagram of a 50-nm-thick FCGT(**B**) and 100-nm-thick (**D**) nanoflake as a function of *D* and *K*. The red dashed line follows the solution of $\sigma_w = 0$.

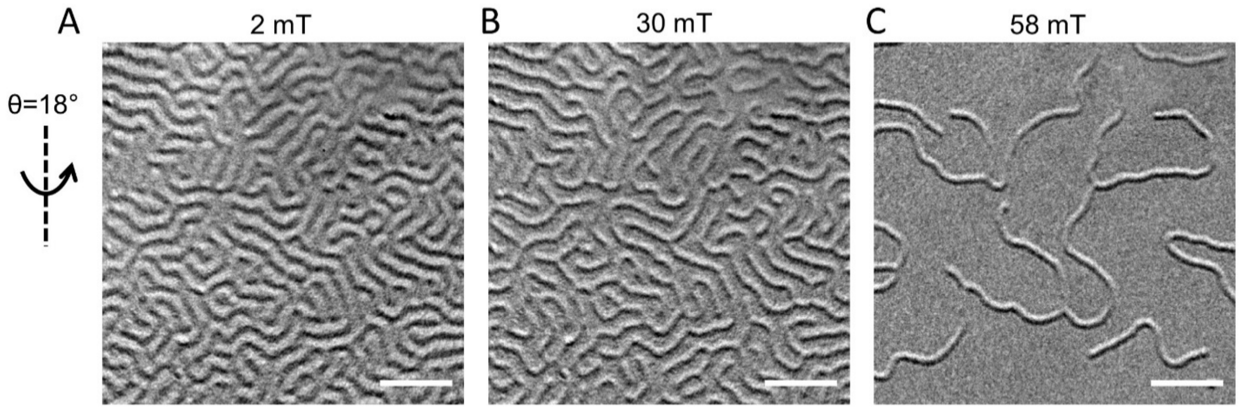

Fig. S12 Evolution of magnetic textures of the 60-nm-thick FCGT nanoflake. Lorentz TEM images of the magnetic textures acquired under applied fields of (**A**) 2 mT, (**B**) 30 mT, and (**C**) 58 mT, with a sample tilt of 18°. Scale bars, 1 μm.

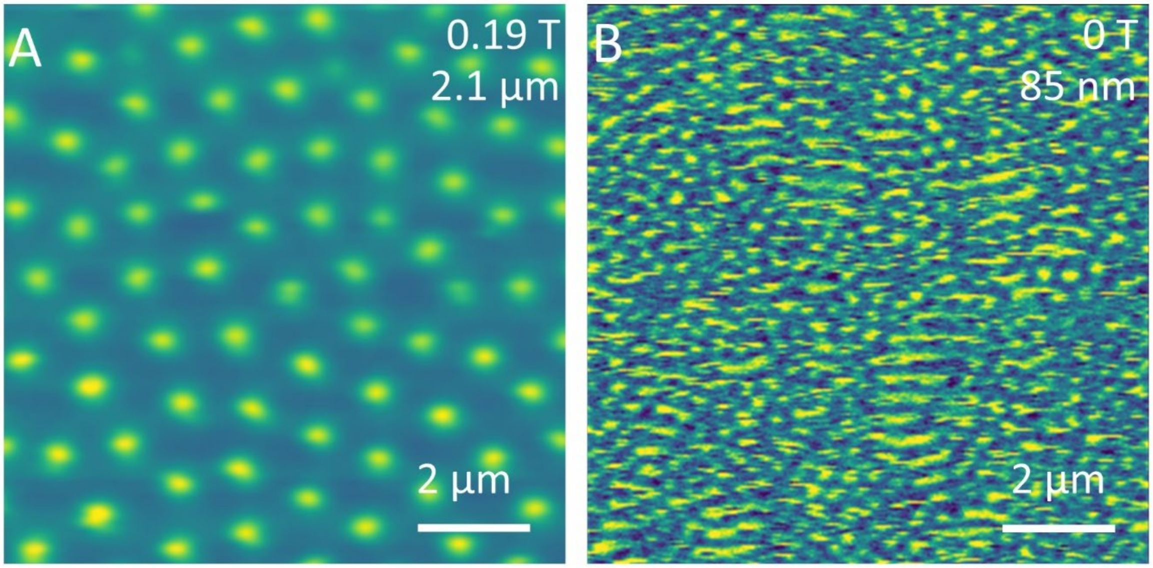

Fig. S13 Room-temperature MFM image in thick nanoflake. (**A**) The skyrmion lattice can be stabilized in the 2.1-µm-thick nanoflake at room temperature and 0.19 T field. (**B**) The MFM image in the 85-nm-thick nanoflake at room temperature and zero field.

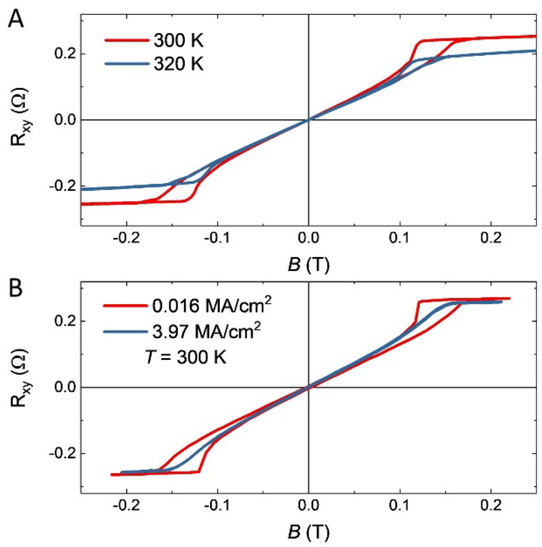

Fig. S14 Elimination of Joule heating effect. (A) Hall measurement at 300 K and 320 K with applied DC current density of 0.016 MA/cm^2. The anomalous Hall resistance is ~0.25 Ω and ~0.21 Ω at 300 K and 320 K, respectively. An obvious hysteresis near the saturated field exists at both temperatures. **(B)** Hall effect at 300 K with applied AC current density of 0.016 MA/cm^{-2} and 3.97 MA/cm^{-2}. The saturated anomalous Hall resistances are almost the same for both current densities. The hysteresis near the saturated field disappeared in the Hall curves measured by large current density. If the Joule heating effect exists, as the current density increases, the saturated anomalous Hall resistance will decrease, and the hysteresis should still be there, similar to the temperature dependence of anomalous Hall effect curves. Thus, the suppression in the Hall effect curve is attributed to current-induced dynamic skyrmions.

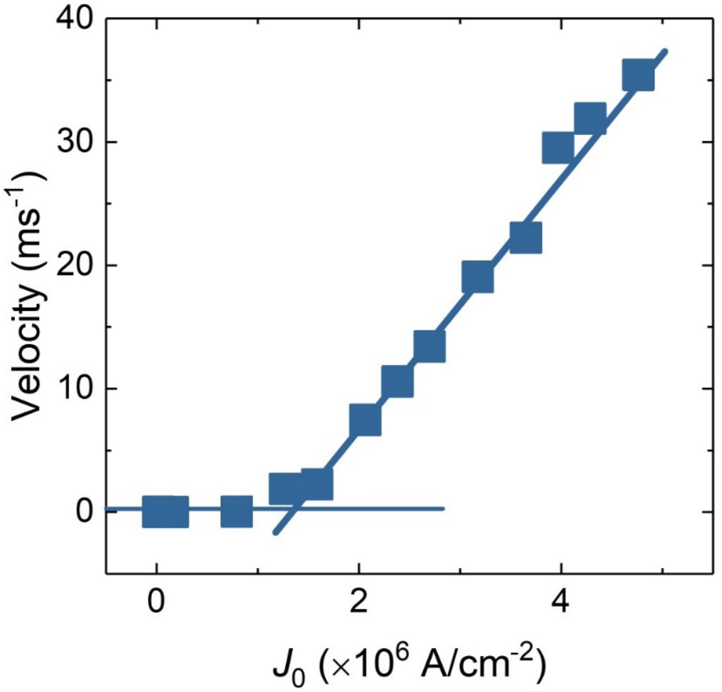

Fig. S15 Estimation of velocity. The skyrmion velocity of the FCGT nanoflake versus current density at room temperature.

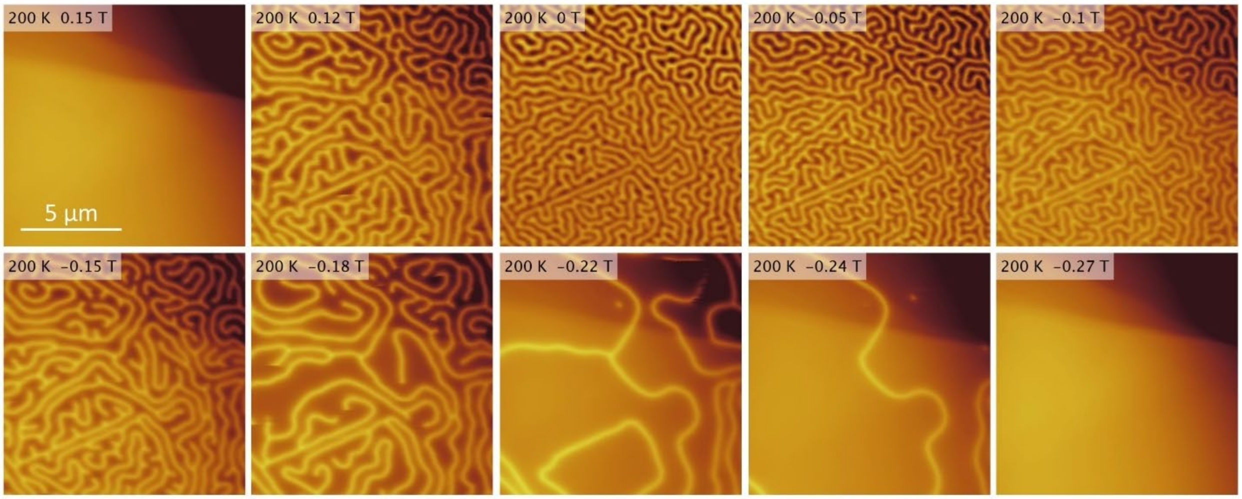

Fig. S16 MFM measurements at 200 K. Field-dependent MFM images on the 261-nm-thick nanoflake at 200 K starting from saturation at 0.15 T. These images were all taken at the same location of the sample. The magnetic contrast in the saturation state is caused by the stray field of the steps one the sample surface. The lift height is 150 nm, and the scale bar is 5 μm.

Movie S1 Magnetic field-dependent magnetic domain. The magnetic domain evolution by the magnetic field at room temperature in a 110-nm-thick nanoflake.

REFERENCES AND NOTES

1. K. F. Mak, J. Shan, D. C. Ralph, Probing and controlling magnetic states in 2D layered magnetic materials. *Nat. Rev. Phys.* **1**, 646–661 (2019).

2. K. S. Burch, D. Mandrus, J.-G. Park, Magnetism in two-dimensional van der Waals materials. *Nature* **563**, 47–52 (2018).

3. Y. Deng, Y. Yu, Y. Song, J. Zhang, N.Z. Wang, Z. Sun, Y. Yi, Y.Z. Wu, S. Wu, J. Zhu, J. Wang, X.H. Chen, Y. Zhang, Gate-tunable room-temperature ferromagnetism in two-dimensional Fe_3GeTe_2. *Nature* **563**, 94–99 (2018).

4. Z. Fei, B. Huang, P. Malinowski, W. Wang, T. Song, J. Sanchez, W. Yao, D. Xiao, X. Zhu, A. F. May, W. Wu, D. H. Cobden, J.H. Chu, X. Xu, Two-dimensional itinerant ferromagnetism in atomically thin Fe_3GeTe_2. *Nat. Mater.* **17**, 778–782 (2018).

5. M. Gibertini, M. Koperski, A. F. Morpurgo, K. S. Novoselov, Magnetic 2D materials and heterostructures. *Nat. Nanotech.* **14**, 408–419 (2019).

6. S. Mühlbauer, B. Binz, F. Jonietz, C. Pfleiderer, A. Rosch, A. Neubauer, R. Georgii, P. Boni, Skyrmion lattice in a chiral magnet. *Science* **323**, 915–919 (2009).

7. X. Z. Yu, Y. Onose, N. Kanazawa, J. H. Park, J. H. Han, Y. Matsui, N. Nagaosa, Y. Tokura, Real-space observation of a two-dimensional skyrmion crystal. *Nature* **465**, 901–904 (2010).

8. K. Karube, J. S. White, N. Reynolds, J. L. Gavilano, H. Oike, A. Kikkawa, F. Kagawa, Y. Tokunaga, H. M. Rønnow, Y. Tokura, Y. Taguchi, Robust metastable skyrmions and their triangular-square lattice structural transition in a high-temperature chiral magnet. *Nat. Mater.* **15**, 1237–1242 (2016).

9. I. Kezsmarki, S. Bordacs, P. Milder, E. Neuber, L. M. Eng, J. S. White, H. M. Ronnow, C. D. Dewhurst, M. Mochizuki, K. Yanai, H. Nakamura, D. Ehlers, V. Tsurkan, A. Loidl, Néel-type skyrmion lattice with confined orientation in the polar magnetic semiconductor GaV_4S_8. *Nat. Mater.* **14**, 1116–1122 (2015).

10. T. Kurumaji, T. Nakajima, V. Ukleev, A. Feoktystov, T.H. Arima, K. Kakurai, Y. Tokura, Néel-Type skyrmion lattice in the tetragonal polar magnet $VOSe_2O_5$. *Phys. Rev. Lett.* **119**, 237201 (2017).

11. S. Heinze, K. von Bergmann, M. Menzel, J. Brede, A. Kubetzka, R. Wiesendanger, G. Bihlmayer, S. Blügel, Spontaneous atomic-scale magnetic skyrmion lattice in two dimensions. *Nat. Phys.* **7**, 713–718 (2011).
12. N. Romming, C. Hanneken, M. Menzel, J. E. Bickel, B. Wolter, K. von Bergmann, A. Kubetzka, R. Wiesendanger, Writing and deleting single magnetic skyrmions. *Science* **341**, 636–639 (2013).
13. S. Woo, K. Litzius, B. Krüger, M.Y. Im, L. Caretta, K. Richter, M. Mann, A. Krone, R. M. Reeve, M. Weigand, P. Agrawal, I. Lemesh, M. A. Mawass, P. Fischer, M. Kläui, G. S. D. Beach, Observation of room-temperature magnetic skyrmions and their current-driven dynamics in ultrathin metallic ferromagnets. *Nat. Mater.* **15**, 501–506 (2016).
14. R. Chen, Y. Gao, X. Zhang, R. Zhang, S. Yin, X. Chen, X. Zhou, Y. Zhou, J. Xia, Y. Zhou, S. Wang, F. Pan, Y. Zhang, C. Song, Realization of isolated and high-density skyrmions at room temperature in uncompensated synthetic antiferromagnets. *Nano Lett.* **20**, 3299–3305 (2020).
15. B. Ding, Z. Li, G. Xu, H. Li, Z. Hou, E. Liu, X. Xi, F. Xu, Y. Yao, W. Wang, Observation of magnetic skyrmion bubbles in a van der Waals ferromagnet Fe_3GeTe_2. *Nano Lett.* **20**, 868–873 (2020).
16. M.-G. Han, J. A. Garlow, Y. Liu, H. Zhang, J. Li, D. DiMarzio, M. W. Knight, C. Petrovic, D. Jariwala, Y. Zhu, Topological magnetic-spin textures in two-dimensional van der Waals $Cr_2Ge_2Te_6$. *Nano Lett.* **19**, 7859–7865 (2019).
17. Y. Wu, S. Zhang, J. Zhang, W. Wang, Y. L. Zhu, J. Hu, G. Yin, K. Wong, C. Fang, C. Wan, X. Han, Q. Shao, T. Taniguchi, K. Watanabe, J. Zang, Z. Mao, X. Zhang, K. L. Wang, Néel-type skyrmion in WTe_2/Fe_3GeTe_2 van der Waals heterostructure. *Nat. Commun.* **11**, 3860 (2020).
18. T.-E. Park, L. Peng, J. Liang, A. Hallal, F. S. Yasin, X. Zhang, K. M. Song, S. J. Kim, K. Kim, M. Weigand, G. Schütz, S. Finizio, J. Raabe, K. Garcia, J. Xia, Y. Zhou, M. Ezawa, X. Liu, J. Chang, H. C. Koo, Y. D. Kim, M. Chshiev, A. Fert, H. Yang, X. Yu, S. Woo, Néel-type skyrmions and their current-induced motion in van der Waals ferromagnet-based heterostructures. *Phys. Rev. B* **103**, 104410 (2021).
19. M. Yang, Q. Li, R. V. Chopdekar, R. Dhall, J. Turner, J. D. Carlström, C. Ophus, C. Klewe, P. Shafer, A. T. N'Diaye, J. W. Choi, G. Chen, Y. Z. Wu, C. Hwang, F. Wang, Z. Q. Qiu, Creation of skyrmions in van der Waals ferromagnet Fe_3GeTe_2 on $(Co/Pd)_n$ superlattice. *Sci. Adv.* **6**, eabb5157 (2020).

20. A. F. May, D. Ovchinnikov, Q. Zheng, R. Hermann, S. Calder, B. Huang, Z. Fei, Y. Liu, X. Xu, M. A. McGuire, Ferromagnetism near room temperature in the cleavable van der Waals crystal Fe_5GeTe_2. *ACS Nano* **13**, 4436–4442 (2019).
21. H. Zhang, R. Chen, K. Zhai, X. Chen, L. Caretta, X. Huang, R.V. Chopdekar, J. Cao, J. Sun, J. Yao, R. Birgeneau, R. Ramesh, Itinerant ferromagnetism in van der Waals $Fe_{5-x}GeTe_2$ crystals above room temperature. *Phys. Rev. B* **102**, 064417 (2020).
22. A. F. May, M.-H. Du, V. R. Cooper, M. A. McGuire, Tuning magnetic order in the van der Waals metal Fe_5GeTe_2 by cobalt substitution. *Phys. Rev. Mater.* **4**, 074008 (2020).
23. C. Tian, F. Pan, S. Xu, K. Ai, T. Xia, P. Cheng, Tunable magnetic properties in van der Waals crystals $(Fe_{1-x}Co_x)_5GeTe_2$. *Appl. Phys. Lett.* **116**, 202402 (2020).
24. H.-J. Deiseroth, K. Aleksandrov, C. Reiner, L. Kienle, R. K. Kremer, Fe_3GeTe_2 and Ni_3GeTe_2 – Two new layered transition-metal compounds: Crystal structures, HRTEM investigations, and magnetic and electrical properties. *Eur. J. Inorg. Chem.* **2006**, 1561–1567 (2006).
25. J. Seo, D. Y. Kim, E. S. An, K. Kim, G.-Y. Kim, S.-Y. Hwang, D. W. Kim, B. G. Jang, H. Kim, G. Eom, S. Y. Seo, R. Stania, M. Muntwiler, J. Lee, K. Watanabe, T. Taniguchi, Y. J. Jo, J. Lee, B. I. Min, M. H. Jo, H. W. Yeom, S.-Y. Choi, J. H. Shim, J. S. Kim, Nearly room temperature ferromagnetism in a magnetic metal-rich van der Waals metal. *Sci. Adv.* **6**, eaay8912 (2020).
26. S. Laref, K.-W. Kim, A. Manchon, Elusive Dzyaloshinskii-Moriya interaction in monolayer Fe_3GeTe_2. *Phys. Rev. B* **102**, 060402 (2020).
27. T. Moriya, Anisotropic superexchange interaction and weak ferromagnetism. *Phys. Rev.* **120**, 91–98 (1960).
28. A. N. Bogdanov, D. A. Yablonskii, Thermodynamically stable `vortices' in magnetically ordered crystals. The mixed state of magnets. *Zh. Eksp. Teor. Fiz.* **95**, 178–182 (1989).
29. A. N. Bogdanov, A. Hubert, The properties of isolated magnetic vortices. *Phys. Stat. Sol.* **186**, 527–543 (1994).
30. M. J. Benitez, A. Hrabec, A. P. Mihai, T. A. Moore, G. Burnell, D. McGrouther, C. H. Marrows, S. McVitie, Magnetic microscopy and topological stability of homochiral Néel domain walls in a Pt/Co/AlO_x trilayer. *Nat. Commun.* **6**, 8957 (2015).

31. S. D. Pollard, J. A. Garlow, J. Yu, Z. Wang, Y. Zhu, H. Yang, Observation of stable Néel skyrmions in cobalt/palladium multilayers with Lorentz transmission electron microscopy. *Nat. Commun.* **8**, 14761 (2017).
32. R. Streubel, C.H. Lambert, N. Kent, P. Ercius, A. T. N'Diaye, C. Ophus, S. Salahuddin, P. Fischer, Experimental evidence of chiral ferrimagnetism in amorphous GdCo films. *Adv. Mater.* **30**, 1800199 (2018).
33. M. W. Tate, P. Purohit, D. Chamberlain, K. X. Nguyen, R. Hovden, C. S. Chang, P. Deb, E. Turgut, J. T. Heron, D. G. Schlom, D. C. Ralph, G. D. Fuchs, K. S. Shanks, H. T. Philipp, D. A. Muller, S. M. Gruner, High dynamic range pixel array detector for scanning transmission electron microscopy. *Microsc. Microanal.* **22**, 237–249 (2016).
34. T. Denneulin, J. Caron, M. Hoffmann, M. Lin, H. K. Tan, A. Kovacs, S. Blugel, R. E. Dunin-Borkowski, Off-axis electron holography of Néel-type skyrmions in multilayers of heavy metals and ferromagnets. *Ultramicroscopy* **220**, 113155 (2021).
35. X. Z. Yu, N. Kanazawa, Y. Onose, K. Kimoto, W. Z. Zhang, S. Ishiwata, Y. Matsui, Y. Tokura, Near room-temperature formation of a skyrmion crystal in thin-films of the helimagnet FeGe. *Nat. Mater.* **10**, 106–109 (2011).
36. F. Zheng, F. N. Rybakov, A. B. Borisov, D. Song, S. Wang, Z.A. Li, H. du, N. S. Kiselev, J. Caron, A. Kovács, M. Tian, Y. Zhang, S. Blügel, R. E. Dunin-Borkowski, Experimental observation of chiral magnetic bobbers in B20-type FeGe. *Nat. Nanotech.* **13**, 451–455 (2018).
37. A. K. Srivastava, P. Devi, A. K. Sharma, T. Ma, H. Deniz, H. L. Meyerheim, C. Felser, S. S. P. Parkin, Observation of robust Néel skyrmions in metallic PtMnGa. *Adv. Mater.* **32**, 1904327 (2020).
38. M. Baćani, M. A. Marioni, J. Schwenk, H. J. Hug, How to measure the local Dzyaloshinskii-Moriya interaction in skyrmion thin-film multilayers. *Sci. Rep.* **9**, 3114 (2019).
39. Y. Guang, I. Bykova, Y. Liu, G. Yu, E. Goering, M. Weigand, J. Gräfe, S. K. Kim, J. Zhang, H. Zhang, Z. Yan, C. Wan, J. Feng, X. Wang, C. Guo, H. Wei, Y. Peng, Y. Tserkovnyak, X. Han, G. Schütz, Creating zero-field skyrmions in exchange-biased multilayers through X-ray illumination. *Nat. Commun.* **11**, 949 (2020).
40. S. Zhang, J. Zhang, Q. Zhang, C. Barton, V. Neu, Y. Zhao, Z. Hou, Y. Wen, C. Gong, O. Kazakova, W. Wang, Y. Peng, D. A. Garanin, E. M. Chudnovsky, X. Zhang, Direct writing of

room temperature and zero field skyrmion lattices by a scanning local magnetic field. *Appl. Phys. Lett.* **112**, 132405 (2018).

41. A. V. Ognev, A. G. Kolesnikov, Y. J. Kim, I. H. Cha, A. V. Sadovnikov, S. A. Nikitov, I. V. Soldatov, A. Talapatra, J. Mohanty, M. Mruczkiewicz, Y. Ge, N. Kerber, F. Dittrich, P. Virnau, M. Kläui, Y. K. Kim, A. S. Samardak, Magnetic direct-write skyrmion nanolithography. *ACS Nano* **14**, 14960–14970 (2020).

42. H. Oike, A. Kikkawa, N. Kanazawa, Y. Taguchi, M. Kawasaki, Y. Tokura, F. Kagawa, Interplay between topological and thermodynamic stability in a metastable magnetic skyrmion lattice. *Nat. Phys.* **12**, 62–66 (2016).

43. I. Lemesh, K. Litzius, M. Böttcher, P. Bassirian, N. Kerber, D. Heinze, J. Zázvorka, F. Büttner, L. Caretta, M. Mann, M. Weigand, S. Finizio, J. Raabe, M.Y. Im, H. Stoll, G. Schütz, B. Dupé, M. Kläui, G. S. D. Beach, Current-induced skyrmion generation through morphological thermal transitions in chiral ferromagnetic heterostructures. *Adv. Mater.* **30**, 1805461 (2018).

44. Z. Wang, M. Guo, H.A. Zhou, L. Zhao, T. Xu, R. Tomasello, H. Bai, Y. Dong, S.G. Je, W. Chao, H.S. Han, S. Lee, K.S. Lee, Y. Yao, W. Han, C. Song, H. Wu, M. Carpentieri, G. Finocchio, M.Y. Im, S.Z. Lin, W. Jiang, Thermal generation, manipulation and thermoelectric detection of skyrmions. *Nat. Electron.* **3**, 672–679 (2020).

45. A. Casiraghi, H. Corte-León, M. Vafaee, F. Garcia-Sanchez, G. Durin, M. Pasquale, G. Jakob, M. Kläui, O. Kazakova, Individual skyrmion manipulation by local magnetic field gradients. *Commun. Phys.* **2**, 145 (2019).

46. A. P. Malozemoff, J. C. Slonczewski, *Magnetic Domain Walls in Bubble Materials: Advances in Materials and Device Research* (Academic Press, 2016), vol. 1.

47. W. Wang, M. W. Daniels, Z. Liao, Y. Zhao, J. Wang, G. Koster, G. Rijnders, C.Z. Chang, D. Xiao, W. Wu, Spin chirality fluctuation in two-dimensional ferromagnets with perpendicular magnetic anisotropy. *Nat. Mater.* **18**, 1054–1059 (2019).

48. T. Schulz, R. Ritz, A. Bauer, M. Halder, M. Wagner, C. Franz, C. Pfleiderer, K. Everschor, M. Garst, A. Rosch, Emergent electrodynamics of skyrmions in a chiral magnet. *Nat. Phys.* **8**, 301–304 (2012).

49. D. Liang, J. P. DeGrave, M. J. Stolt, Y. Tokura, S. Jin, Current-driven dynamics of skyrmions stabilized in MnSi nanowires revealed by topological Hall effect. *Nat. Commun.* **6**, 8217 (2015).

50. J. Zang, M. Mostovoy, J. H. Han, N. Nagaosa, Dynamics of skyrmion crystals in metallic thin films. *Phys. Rev. Lett.* **107**, 136804 (2011).
51. M. Raju, A. Yagil, A. Soumyanarayanan, A. K. C. Tan, A. Almoalem, F. Ma, O. M. Auslaender, C. Panagopoulos, The evolution of skyrmions in Ir/Fe/Co/Pt multilayers and their topological Hall signature. *Nat. Commun.* **10**, 696 (2019).
52. T. Yokouchi, S. Hoshino, N. Kanazawa, A. Kikkawa, D. Morikawa, K. Shibata, T.-H. Arima, Y. Taguchi, F. Kagawa, N. Nagaosa, Y. Tokura, Current-induced dynamics of skyrmion strings. *Sci. Adv.* **4**, eaat1115 (2018).
53. R. Aoki, Y. Kousaka, Y. Togawa, Anomalous nonreciprocal electrical transport on chiral magnetic order. *Phys. Rev. Lett.* **122**, 057206 (2019).
54. Y. Tokura, N. Nagaosa, Nonreciprocal responses from non-centrosymmetric quantum materials. *Nat. Commun.* **9**, 3740 (2018).
55. S. S. P. Parkin, M. Hayashi, L. Thomas, Magnetic domain-wall racetrack memory. *Science* **320**, 190–194 (2008).
56. W. Legrand, D. Maccariello, N. Reyren, K. Garcia, C. Moutafis, C. Moreau-Luchaire, S. Collin, K. Bouzehouane, V. Cros, A. Fert, Room-temperature current-induced generation and motion of sub-100 nm skyrmions. *Nano Lett.* **17**, 2703–2712 (2017).
57. R. Juge, S.G. Je, D.S. Chaves, L.D. Buda-Prejbeanu, J. Peña-Garcia, J. Nath, I.M. Miron, K.G. Rana, L. Aballe, M. Foerster, F. Genuzio, T.O. Menteş, A. Locatelli, F. Maccherozzi, S.S. Dhesi, M. Belmeguenai, Y. Roussigné, S. Auffret, S. Pizzini, G. Gaudin, J. Vogel, O. Boulle, Current-driven skyrmion dynamics and drive-dependent skyrmion Hall effect in an ultrathin film. *Phys. Rev. Appl.* **12**, 044007 (2019).
58. K. Litzius, J. Leliaert, P. Bassirian, D. Rodrigues, S. Kromin, I. Lemesh, J. Zazvorka, K.J. Lee, J. Mulkers, N. Kerber, D. Heinze, N. Keil, R. M. Reeve, M. Weigand, B. van Waeyenberge, G. Schütz, K. Everschor-Sitte, G. S. D. Beach, M. Kläui, The role of temperature and drive current in skyrmion dynamics. *Nat. Electron.* **3**, 30–36 (2020).
59. M. Raju, A. P. Petrović, A. Yagil, K. S. Denisov, N. K. Duong, B. Göbel, E. Şaşıoğlu, O. M. Auslaender, I. Mertig, I. V. Rozhansky, C. Panagopoulos, Colossal topological Hall effect at the transition between isolated and lattice-phase interfacial skyrmions. *Nat. Commun.* **12**, 2758 (2021).

60. A. Soumyanarayanan, M. Raju, A. L. Gonzalez Oyarce, A. K. C. Tan, M.Y. Im, A. P. Petrović, P. Ho, K. H. Khoo, M. Tran, C. K. Gan, F. Ernult, C. Panagopoulos, Tunable room-temperature magnetic skyrmions in Ir/Fe/Co/Pt multilayers. *Nat. Mater.* **16**, 898–904 (2017).
61. A. B. Yankovich, C. Zhang, A. Oh, T. J. A. Slater, F. Azough, R. Freer, S. J. Haigh, R. Willett, P. M. Voyles, Non-rigid registration and non-local principle component analysis to improve electron microscopy spectrum images. *Nanotechnology* **27**, 364001 (2016).
62. A. Vansteenkiste, J. Leliaert, M. Dvornik, M. Helsen, F. Garcia-Sanchez, B. van Waeyenberge, The design and verification of MuMax3. *AIP Adv.* **4**, 107133 (2014).
63. Z. Malek, V. Kambersky, On the theory of the domain structure of thin films of magnetically uni-axial materials. *Czeck. J. Phys.* **8**, 416–421 (1958).
64. S. Woo, K. M. Song, X. Zhang, Y. Zhou, M. Ezawa, X. Liu, S. Finizio, J. Raabe, N. J. Lee, S.I. Kim, S.Y. Park, Y. Kim, J.Y. Kim, D. Lee, O.J. Lee, J. W. Choi, B.C. Min, H. C. Koo, J. Chang, Current-driven dynamics and inhibition of the skyrmion Hall effect of ferrimagnetic skyrmions in GdFeCo films. *Nat. Commun.* **9**, 959 (2018).
65. M. Mansuripur, Computation of electron diffraction patterns in Lorentz electron microscopy of thin magnetic films. *J. Appl. Phys.* **69**, 2455–2464 (1991).
66. T. Xu, Z. Chen, H.A. Zhou, Z. Wang, Y. Dong, L. Aballe, M. Foerster, P. Gargiani, M. Valvidares, D.M. Bracher, T. Savchenko, A. Kleibert, R. Tomasello, G. Finocchio, S.G. Je, M.Y. Im, D.A. Muller, W. Jiang, Imaging the spin chirality of ferrimagnetic Néel skyrmions stabilized on topological antiferromagnetic Mn_3Sn. *Phys. Rev. Mater.* **5**, 084406 (2021).
67. N. Romming, A. Kubetzka, C. Hanneken, K. von Bergmann, R. Wiesendanger, Field-dependent size and shape of single magnetic skyrmions. *Phys. Rev. Lett.* **114**, 177203 (2015).
68. P. Huang, T. Schönenberger, M. Cantoni, L. Heinen, A. Magrez, A. Rosch, F. Carbone, H. M. Rønnow, Melting of a skyrmion lattice to a skyrmion liquid via a hexatic phase. *Nat. Nanotechnol.* **15**, 761–767 (2020).
69. J. Zázvorka, F. Dittrich, Y. Ge, N. Kerber, K. Raab, T. Winkler, K. Litzius, M. Veis, P. Virnau, M. Kläui, Skyrmion lattice phases in thin film multilayer. *Adv. Funct. Mater.* **30**, 2004037 (2020).
